# Syntheses and chemical properties of β-nicotinamide riboside and its analogues and derivatives

**DOI:** 10.3762/bjoc.15.36

**Published:** 2019-02-13

**Authors:** Mikhail V Makarov, Marie E Migaud

**Affiliations:** 1 Mitchell Cancer Institute, University of South Alabama, 1660 Springhill Ave., Mobile, AL 36604, USA

**Keywords:** anomers, glycosylation, isotopologues, isotopomeres, nicotinamide riboside

## Abstract

The β-anomeric form of nicotinamide riboside (NR^+^) is a precursor for nicotinamide adenine dinucleotide (NAD^+^), a redox cofactor playing a critical role in cell metabolism. Recently, it has been demonstrated that its chloride salt (NR^+^Cl^−^) has beneficial effects, and now NR^+^Cl^−^ is available as a dietary supplement. Syntheses and studies of analogues and derivatives of NR^+^ are of high importance to unravel the role of NR^+^ in biochemical processes in living cells and to elaborate the next generation of NR^+^ derivatives and conjugates with the view of developing novel drug and food supplement candidates. This review provides an overview of the synthetic approaches, the chemical properties, and the structural and functional modifications which have been undertaken on the nicotinoyl riboside scaffold.

## Review

### 1. Introduction

**Figure 1:**
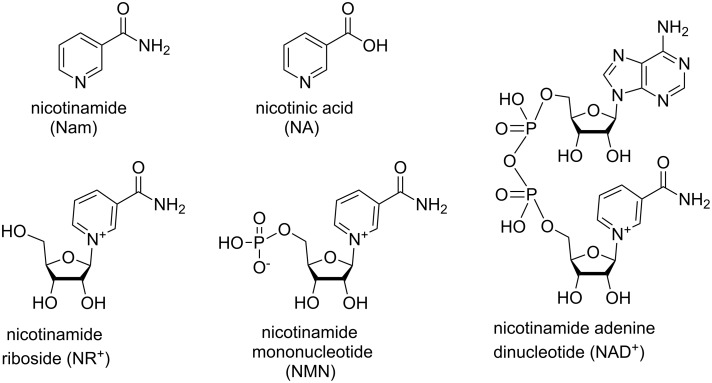
Structural formulas of Nam, NA, NR^+^, NMN, and NAD^+^.

1-(β-D-Ribofuranosyl)nicotinamide (also referred to as nicotinamide riboside, NR^+^) is one of the multiple precursors of nicotinamide adenine dinucleotide (NAD^+^, Figure 1), an intracellular redox cofactor, central to all cells’ biochemistry. Unlike its phosphorylated and reduced forms, NAD^+^ is also a non-redox substrate for many intra- and extracellular enzymes, participating in cellular signaling pathways and regulating post-translational protein modification events [1–3]. During the process of aging and in several disease models, the demand on the NAD^+^ pool far exceeds its availability [4–6]. While nicotinic acid [7] and nicotinamide [8–9] are long-standing vitamin B3 supplements known to restore depleted NAD^+^ levels [10–12], it was only recently that NR^+^ and its phosphorylated form, nicotinamide mononucleotide (NMN), have become orally available as nutraceutical precursors of NAD^+^ [13–16]. This followed the identification of the biosynthetic pathways which use these ribosylated building blocks for the efficient synthesis of NAD^+^ in humans. Unlike other nucleosides, ribosylated nicotinamide displays a relatively labile glycosidic bond, which renders its synthesis and its manipulation challenging. Yet, modifications of NR^+^ through the introduction of appendages and synthesis of structural analogues open numerous possibilities for the creation and development of novel drug-like candidates that may stimulate, inhibit or interfere with cellular processes in cells, where metabolism and cellular signaling crucially rely on a constant supply of NAD^+^. Because of the commercial nature and physiological relevance of the NR^+^ scaffold, many reports of its synthesis have been made through patents and biology-focused publications rather than standard chemistry publications. The present review summarizes the chemistry which has been explored in the generation of NR^+^, NMN and their derivatives throughout the field.

### 2. Synthesis of β-nicotinamide riboside

**Figure 2:**
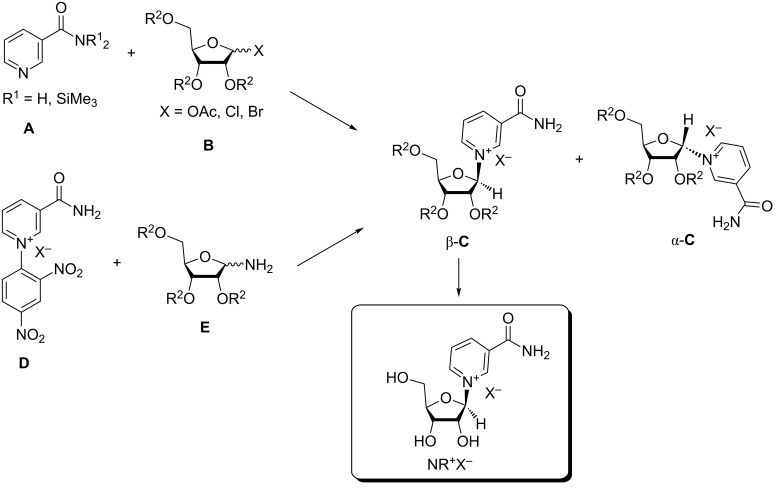
Main synthetic routes to nicotinamide riboside (NR^+^X^−^).

Most synthetic routes to nicotinamide riboside salt forms (NR^+^X^−^) may be subdivided into two main categories (Figure 2). One is via a reaction between nicotinamide (Nam) or its analogues or derivatives **A** and a peracylated (halo)-D-ribofuranose **B** resulting in acylated intermediate **C** that is subsequently converted into desired NR^+^X^−^, the other is via a condensation of *N*-(2,4-dinitrophenyl)-3-carbamoylpyridinium salt **D** with derivatives of D-ribofuranosylamine **E** [17–18]. To date, the second approach has been far less exploited and remains with limited applicability, while most interesting developments in terms of synthetic efficiency, improved stereoselectivity and overall yields relate to the first approach. This approach, the synthetic glycosylation conditions depend on the nature of the sugar component. These conditions differ whether 1-halo-2,3,5-tri-*O*-acyl- or 1,2,3,5-tetra-*O*-acyl-D-ribofuranose is used, as fully acylated ribofuranoses require the usage of Friedel–Crafts catalysts to be activated as glycosylation reagents.

As it is shown in Figure 2, reactions between compounds **A** and **B** may result in two anomeric α- and β-forms of nicotinamide riboside. The stereochemical outcomes of the synthesis (anomeric ratio) depends on the nature and the stereochemistry of the leaving group X in sugar **B** (α- or β-anomer), on the nature of the substituents at the amide nitrogen atom in Nam, and on the conditions of glycosylation, such as solvent and temperature. Because only the β-form of NR^+^ is of biochemical and medicinal relevance, any valuable method of synthesis of NR^+^ should offer a high level of β-stereoselectivity. Approaches based on the glycosylation of Nam will be discussed first and will be followed by a review of the NR^+^ syntheses accomplished using *N*-(2,4-dinitrophenyl)-3-carbamoylpyridinium salts and aminosugar precursors. The methods to gain access to the phosphorylated form on NR^+^ will then be discussed within the context of reactivity and chemoselectivity. The last section will address the enzymatic processes which have been adopted to access NR^+^ and NMN.

#### 2.1. Glycosylation of nicotinamide

As mentioned above, glycosylation of Nam may be performed either by using halosugars, i.e., 2,3,5-tri-*O*-acyl-D-ribofuranosyl halides, or by applying 1,2,3,5-tetra-*O*-acyl-D-ribofuranoses. The latter fully acylated sugars require different reaction conditions for the glycosylation of nicotinamide. Historically, 2,3,5-tri-*O*-acyl-D-ribofuranosyl halides were used first (so called the halo route) and will be considered in the first place.

**Scheme 1:**
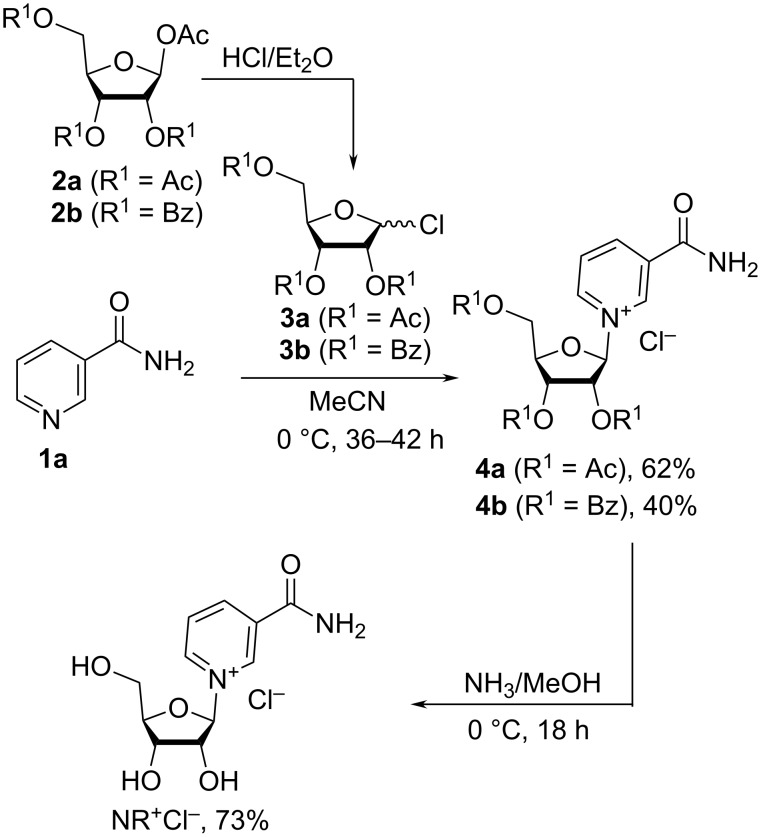
Synthesis of NR^+^Cl^−^ based on the reaction of peracylated chlorosugars with Nam.

**2.1.1. Glycosylation with 2,3,5-tri-*O*-acylribofuranosyl halides:** The first works on the chemical synthesis of nicotinamide riboside salts (NR^+^X^−^) were described by Todd et al. [19–20] in 1950–1957 and were based on the glycosylation of Nam (**1a**) in anhydrous acetonitrile with 2,3,5-tri-*O*-acetyl-D-ribofuranosyl bromide to yield the triacetylated bromide salt, with 2,3,5-tri-*O*-acetyl-D-ribofuranosyl chloride to yield the triacetylated chloride salt or with 2,3,5-tri-*O*-benzoyl-D-ribofuranosyl chloride to yield the tribenzoylated chloride salt. The halosugars were obtained from 1,2,3,5-tetra-*O*-acetyl-D-β-ribofuranose (**2a**) or 1-*O*-acetyl-2,3,5-tri-*O*-benzoyl-D-β-ribofuranose (**2b**) [21]. Both pyridinium riboside anomers were synthesized, with the best β/α-anomer stereoselectivity obtained when the chloride form of the sugars were used as precursors. Thus, the reaction between Nam (**1a**) and 2,3,5-tri-*O*-acetyl-D-ribofuranosyl chloride (**3a**) in acetonitrile at 0 °C yielded the triacetylated product **4a** mainly in the β-anomer form (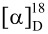 −58.7°) in 62% yield, while the benzoylated analogue **4b** (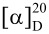 −44.0°) was obtained in lower yields of 40% (Scheme 1). Acetyl and benzoyl protecting groups were then removed from **4a** and **4b** in anhydrous methanol saturated with dry ammonia at 0 °C, to afford NR^+^Cl^−^ (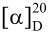 −28.6°) in good yield. The anomeric purity of the acylated intermediates **4a** and **4b** was not reported in [20]. However, these triesters must have contained some admixture of α-anomers as evidenced by the optical rotation of the final product NR^+^Cl^−^. The corresponding nicotinamide mononucleotide (NMN) was prepared from NR^+^Cl^−^ by phosphorylation with phosphoryl chloride in a water/nitromethane solution. When its optical rotation was measured (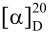 −24°) and compared to a standard solution of NMN generated from NAD^+^, it was concluded that the synthesized NR^+^Cl^−^ consisted of a 4:1 mixture of β- and α-anomers (Scheme 1) [20,22].

With regards to the effect of temperature on the outcomes of the glycosylation step, Atkinson et al. [23] and Jarman et al. [22] pointed out that low temperatures were preferred in order to minimize the rapid decomposition of the α-anomer of tribenzoylated nicotinamide riboside chloride α-**4b** that occurs in the presence of an excess of Nam at 37 °С. Data on the stability of the β-anomer of tribenzoylated nicotinamide riboside chloride under the same conditions were not reported.

**Figure 3:**
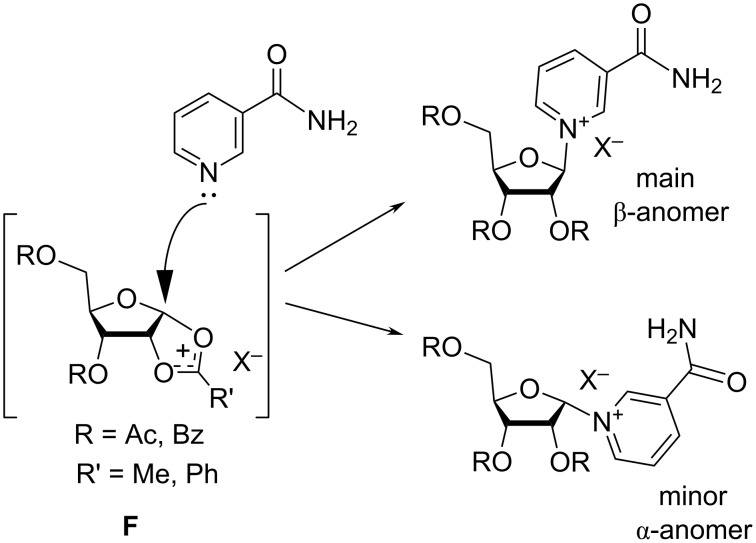
Predominant formation of β-anomer over α-anomer of NR^+^X^−^.

The stereoselectivity of the glycosylation reaction is said to be attributed to the formation of the cationic intermediate **F**, in which the C2′-acyl group participates in delocalization of the carbocation on the C1′ position. Nam approaches and attacks this carbocation from the less sterically hindered face to give the β-nicotinamide riboside derivatives (Figure 3). A very comprehensive and thorough discussion of the glycosylation mechanism is provided in a monograph authored by Vorbrüggen and Ruh-Pohlenz [24].

**Scheme 2:**
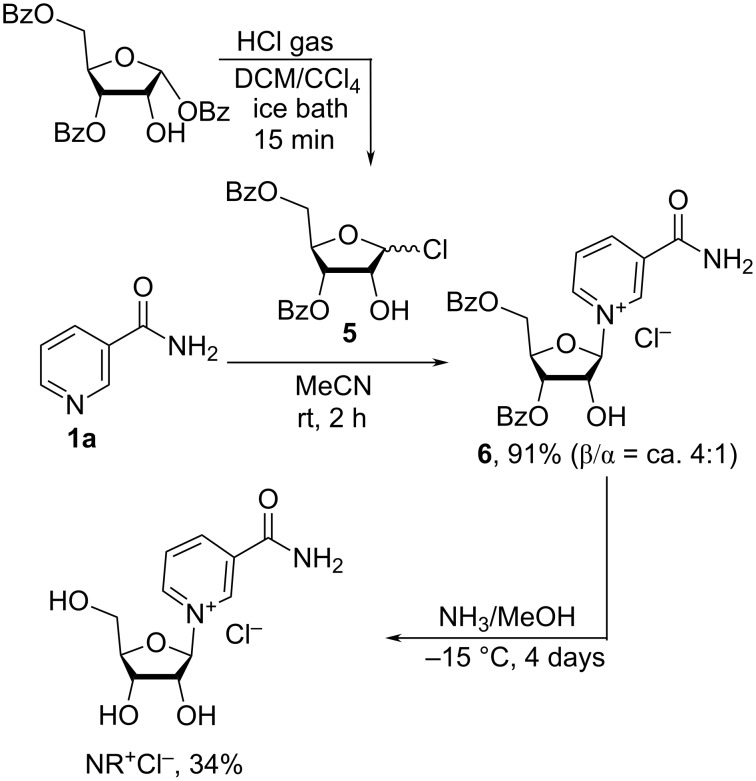
Synthesis of NR^+^Cl^−^ by reacting 3,5-di-*O*-benzoyl-D-ribofuranosyl chloride (**5**) with Nam (**1a**).

Jarman et al. [22] demonstrated that NR^+^Cl^−^ may be prepared from the partially protected crystalline 3,5-di-*O*-benzoyl-D-ribofuranosyl chloride (**5**). Unfortunately, the anomeric α/β ratio was not reported. Once 3,5-di-*O*-benzoyl-D-ribofuranosyl chloride (**5**) had reacted with Nam (**1a**) in acetonitrile, the dibenzoate intermediate **6** was hydrolyzed under anhydrous conditions in methanolic ammonia (Scheme 2).

**Figure 4:**
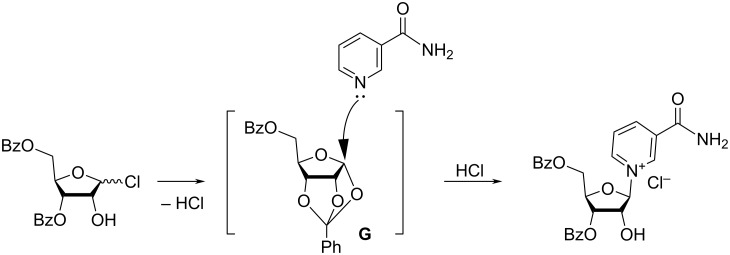
Mechanism of the formation of the β-anomer of the glycosylated product in the case of the reaction of 3,5-di-*O*-benzoyl-α/β-D-ribofuranosyl chloride with Nam.

The 3,5-di-*O*-benzoyl-α/β-D-ribofuranosyl chloride (**5**) was prepared from an anomerically pure 1,3,5-tri-*O*-benzoyl-α-D-ribofuranose. The glycosylation reaction afforded the dibenzoate **6** as a ca. 4:1 mixture of β/α-anomers as determined by ^1^H NMR spectroscopy. In the absence of the anchimeric assistance of the 2′-*O*-acyl group that favours formation of the β-anomer, it was hypothesized [22] that the glycosylation step would proceed through the formation of an orthoester intermediate **G** (Figure 4) which Nam would subsequently attack.

The removal of the benzoyl groups in 1 M methanolic ammonia was conducted at −15 °C for 4 days to afford the nucleoside NR^+^Cl^−^ in the rather low yield of 34% with 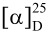 −37°. This optical rotation and ^1^H NMR spectroscopy data indicated that the resulting NR^+^Cl^−^ contained ca. 85% of the desired β-anomer.

A recent patent publication by Migaud et al. [25] described preparing nicotinamide riboside triacetate chloride **4a** from 2,3,5-tri-*O*-acetyl-D-ribofuranosyl chloride. The latter was synthesized from 1-*O*-methyl-2,3,5-tri-*O*-acetyl-D-ribofuranose in 87% yield and contained 6% of α-chlororiboside triacetate. The 2,3,5-tri-*O*-acetyl-1-D-ribofuranosyl chloride was reacted with equimolar amount of Nam in acetonitrile at 70 °C for 20 min resulting in compound **4a** as a ca. 6:4 β/α anomeric mixture. Addition of acetone to the hot reaction mixture immediately upon completion of the glycosylation and subsequent filtration of a precipitated product and repeated washing with acetone made it possible to isolate the anomerically pure β-**4a** as a free-flowing powder, while the α-anomer and other impurities remained in the acetone washings. This patent also divulges the synthesis of 2,3,5-tri-*O*-acetyl-D-ribofuranosyl chloride (β/α ratio is 6:4) using an extruder with the aim of developing a continuous production of compound **4a**. Deacetylation of **4a** to give the desired β-NR^+^Cl^−^ salt was studied under acidic (aq or gas HCl in methanol or ethanol) and (30% aqueous ammonia in methanol at −10 °C) basic conditions. It was demonstrated that the best results in terms of β-NR^+^Cl^−^ purity and yields were achieved when the acetate deprotection was carried under anhydrous conditions using 3 molar equivalents of 1.25 M HCl in methanol. A significant advantage of an acidic methanolic deprotection is the formation of methyl acetate rather than acetamide. The later byproduct has been classified by the International Agency for Research on Cancer (IARC) as a Group 2B compound, i.e., as a possible human carcinogen, and therefore its residual presence in final products should be avoided.

**Scheme 3:**
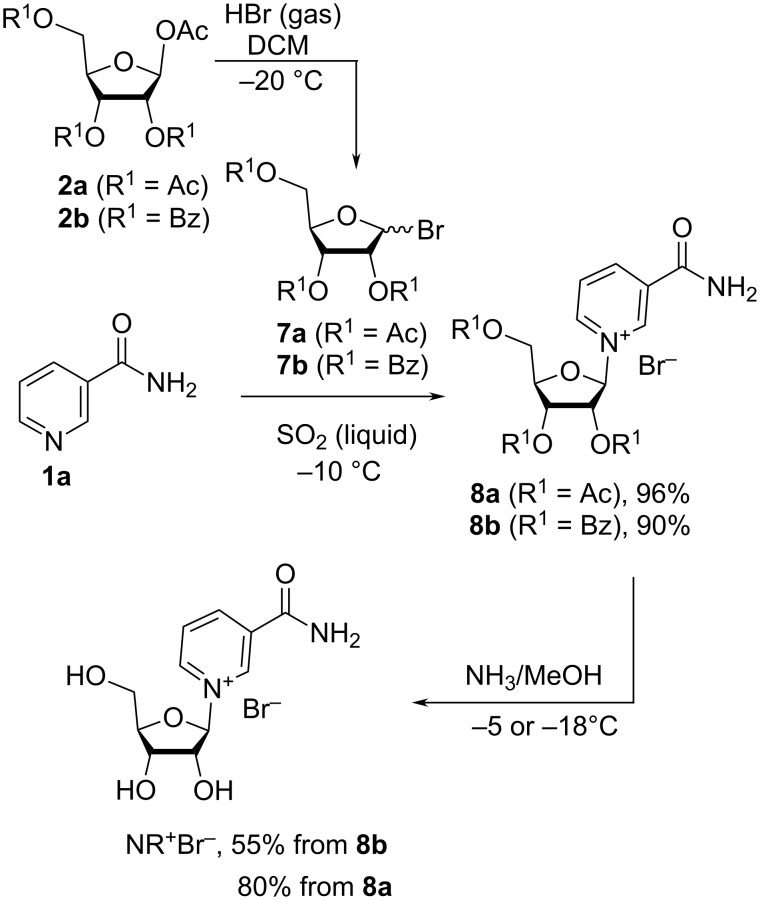
Synthesis of NR^+^Br^−^ by reacting bromosugars with Nam (**1a**).

The yields and the stereoselectivity towards the NR^+^ β-anomer were also improved when both temperature and solvents were optimized and Nam (**1**) was reacted with 2,3,5-tri-*O*-acetyl-D-ribofuranosyl bromide (**7a**) [26–27] or 2,3,5-tri-*O*-benzoyl-D-ribofuranosyl bromide (**7b**) [22,27] (Scheme 3). The bromosugars were generated from tetra-*O*-acetyl-β-D-ribofuranose (**2a**) or 1-*O*-acetyl-2,3,5-tri-*O*-benzoyl-β-D-ribofuranose (**2b**) using gaseous HBr. There, Jarman et al. [22] prepared the anomerically pure benzoyl-protected β-analogue **8b** (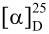 −42.0°) in 61% yield using acetonitrile at −5 °C. Unfortunately, the methods for the deprotection of **8b** were not described. Mikhailopulo et al. [26] studied a range of solvents, such as sulfolane, sulfur dioxide and nitromethane, to improve on the glycosylation reaction outcomes. It was found that yields and anomeric purity were best improved with liquid SO_2_; the yields of **8a** (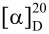 −44.7°) and **8b** (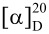 −36.8°) were 96% and 90%, respectively, with the isolated product **8b** containing no α-anomer (according to ^1^H NMR data), as specifically emphasized by the authors. The deprotection of **8b** conducted at −18 °C in cold methanol saturated with ammonia gas for 72 h yielded NR^+^Br^−^ (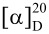 −33.0°) in 55% yield. The loss of the ribosylated species over the course of this type of deprotection highlights that the reactivity towards nucleophiles and Brönsted bases of the C-1′ position in the NR^+^ species is a recurrent issue in NR^+^ chemistry. Finally, Lee et al. [27] studied in detail the regiochemical outcomes of the synthesis of the bromosugars **7a** and **7b** of the glycosylation of Nam (**1a**) with these bromosugars as well as the deprotection of the acylated nucleosides **8a**,**b**. It was observed that the bromination of **2a** with HBr gas in anhydrous DCM gave a 1.5:1 β/α mixture of corresponding anomeric bromides **7a**. This ratio was also observed by Migaud et al. [28] for whom the formation of a 1:1 β/α mixture of these bromides occurred when the tetracetylated ribose **2a** was brominated in dichloromethane with 33% HBr in acetic acid, while a 8:2 β/α mixture was obtained when **2a** was reacted with neat bromotrimethylsilane at room temperature. Bromination of **2b** yielded a 10:1 β/α mixture of the anomeric bromide **7b** [27]. The reaction between Nam (**1a**) and the bromide **7a** was carried out in liquid SO_2_ and proceeded in high yields with the generation of the protected β-NR^+^Br^−^ **8a,** which was isolated by crystallization from a 5:1 acetone/*t*-BuOMe mixture in 90% overall yield from **2a** [27]. Note that dry acetonitrile was also used as a solvent in the glycosylation step instead of liquid SO_2_ but the yields of acetylated NR^+^Br^−^ **8a** were lowered (65%) [27]. Removal of the acetyl groups from **8a** under optimized conditions (3 equivalents of NH_3_, 1 M in MeOH, at −3 to −5 °C for 20 h), followed by crystallization from a 1:5 mixture of MeOH/acetone afforded β-NR^+^Br^−^ as a crystalline solid in high yield (80%) [27]. It should be noted that Lee et al. [27] were not able to reproduce the deprotection of the benzoylated derivative **8b** as described in the work of Mikhailopulo et al. [26]. Instead, they observed a substantial epimerization leading to a ca. 2:1 mixture of the β/α anomers of NR^+^Br^−^ and recommended the use of 1,2,3,5-tetra-*O*-acetyl-β-D-ribofuranose (**2a**) for the synthesis of β-NR^+^Br^−^.

**2.1.2. Glycosylation with 1,2,3,5-tetra-*O*-acyl-ribofuranoses by the silyl-Hilbert–Johnson method:** Further developments in a more efficient and stereoselective synthesis of NR^+^ salt forms were achieved by the application of the silyl-Hilbert–Johnson method based on the glycosylation of silylated heterocyclic bases with acylated (halo)ribofuranoses and Friedel–Crafts catalysts. The main advantage of this method is that the silylation converts polar, often poorly soluble, heterocyclic bases into lipophilic silyl derivatives, resulting in homogeneous reaction mixtures [24]. In the Vorbrüggen version of the silyl Hilbert–Johnson method, trimethylsilyl trifluoromethanesulfonate (TMSOTf) is used as a Friedel–Crafts catalyst.

**Scheme 4:**
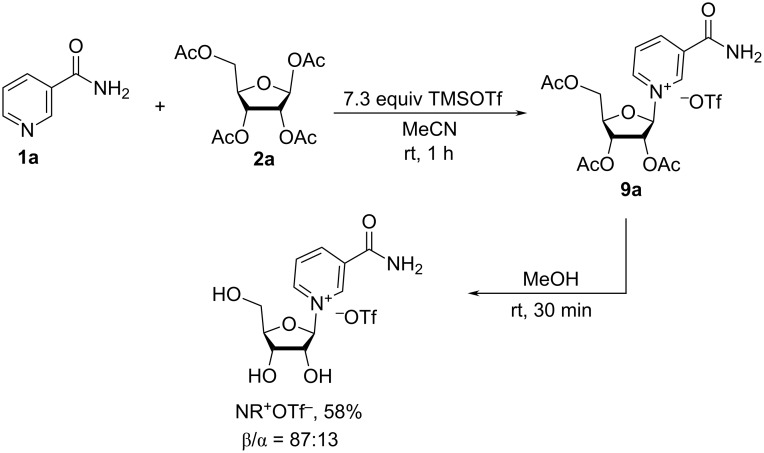
Synthesis of NR^+^OTf^−^ based on the glycosylation of Nam (**1a**) with tetra-*O*-acetyl-β-D-ribofuranose (**2a**) in the presence of TMSOTf.

The TMSOTf-mediated glycosylation of Nam (**1a**) with tetra-*O*-acetyl-β-D-ribofuranose was first proposed by Tanimori et al. in 2002 [29] (Scheme 4). The reaction between Nam (**1a**) and 1,2,3,5-tetra-*O*-acetyl-β-D-ribofuranose (**2a**) in acetonitrile at room temperature resulted in the corresponding triacetate derivative of NR^+^ triflate (OTf^−^) **9a** which was converted without isolation into NR^+^OTf^−^ by methanolysis. The final N-glycoside contained 13% of the α-anomer. This anomer was removed from the crude mixture by chromatography on activated charcoal and crystallization to afford the desired β-NR^+^OTf^−^ in 58% yield. The predominant formation of the β-isomer of NR^+^ is due to the generation of a type **F** cationic intermediate in the course of glycosylation (Figure 3). This intermediate is subsequently approached by Nam from the less hindered side.

**Scheme 5:**
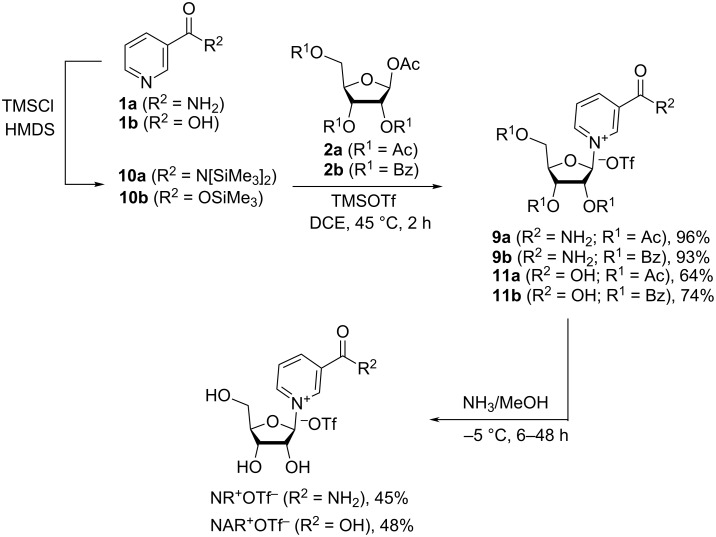
Improved synthesis of NR^+^OTfˉ and NAR^+^OTfˉ based on the glycosylation of pre-silylated Nam or NA with tetra-*O*-acyl-β-D-ribofuranose in the presence of TMSOTf, as adapted from [30].

Subsequently, Franchetti et al. [30] modified the above TMSOTf-protocol by introducing the preliminary silylation of the amide group of Nam, a procedure based on the chemistry developed by Vorbrüggen [24]. This approach was also applied to nicotinic acid (**1b**), for which the corresponding silyl ester **10b** was prepared (Scheme 5) [30]. Nam (**1a**) was silylated to the bis-silylated nicotinamide **10a** using 2 equivalents of TMSCl. The silylation was followed by the reaction of the crude mixture with 1,2,3,5-tetra-*O*-acetyl-β-D-ribofuranose (**2a**) or 1-*O*-acetyl-2,3,5-tri-*O*-benzoyl-β-D-ribofuranose (**2b**) in the presence of TMSOTf in 1,2-dichloroethane solution to give protected NR^+^ derivatives as triflates **9a**,**b**. Critically, a larger excess of TMSCl decreases reaction rates and lowers yields, as the silylation of the N1 atom of Nam occurs under these conditions. Hydrolytic de-esterification of **9a**,**b** was achieved by a treatment with an anhydrous ammonia methanolic solution at −5 °C. Removal of benzoyl groups required prolonged reaction times (48 h versus 6 h for acetyl groups).

**Scheme 6:**
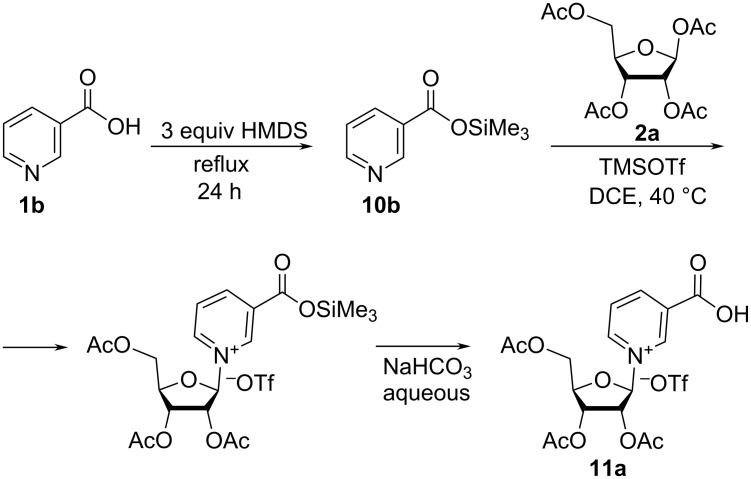
Synthesis of triacetylated NAR^+^OTf^−^ by glycosylation of nicotinic acid trimethylsilyl ester with tetraacetate β-ribose in the presence of TMSOTf.

It should be also noted that in the case of nicotinic acid (**1b**), a modified synthetic protocol was developed, for which silylation was carried out using only 3 equivalents of HMDS in the presence of catalytic amounts of ammonium sulfate without addition of TMSCl [31] (Scheme 6).

**Scheme 7:**
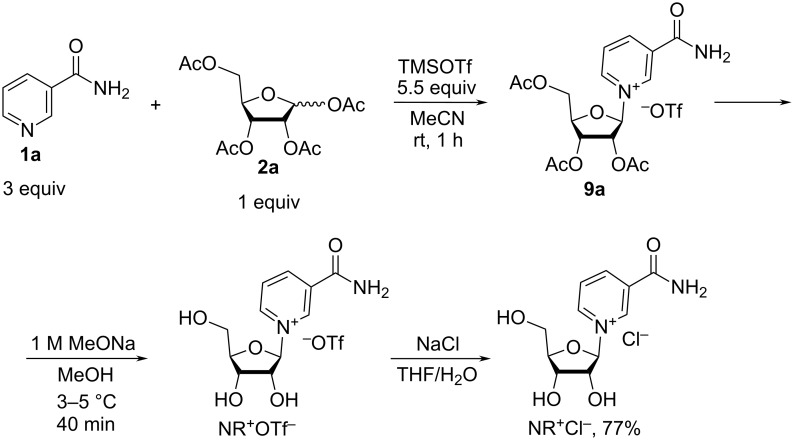
Synthesis of NR^+^Cl^−^ from NR^+^OTf^−^ by means of ion exchange with sodium chloride solution.

It is worth mentioning that, because the triflate salts are not considered as pharmaceutically acceptable in general, the triflate form of NR^+^ must be converted to salts with pharmaceutically acceptable counter anions, such as chloride, before becoming a consumable product. Ion exchange was used by Szczepankiewicz et al. [32] to generate the anomerically pure β-nicotinamide riboside chloride from the triflate salt. The method described in this patent publication is based on the reaction of **1a** with a mixture of α- and β-anomers of 1,2,3,5-tetra-*O*-acetyl-D-ribofuranose (**2a**) in the presence of TMSOTf, with the subsequent removal of the acetyl groups with sodium methoxide in methanol. The triflate removal was conducted by an ion exchange step using a saturated aqueous solution of sodium chloride followed by an extraction with THF to remove the excess of nicotinamide and sodium trifluoromethanesulfonate byproducts (Scheme 7).

The above described procedure uses a 3-fold molar excess of Nam and a 5.5-fold molar excess of TMSOTf compared to α/β-1,2,3,5-tetra-*O*-acetyl-D-ribofuranose (the α/β-ratio is not reported). The need for the removal of the excess reagents renders the isolation procedure cumbersome. Thus, after the first glycosylation step, the reaction mixture was treated with solid sodium hydrocarbonate and diluted with DCM to remove sodium and nicotinamide trifluoromethanesulfonates by filtration. Without further purification, the intermediate **9a** was subjected to hydrolysis in methanolic solution of 1 M sodium methylate at 3–5 °C. The pH was then adjusted to 4 and then to 6–7 and the aqueous solution containing the nicotinamide riboside was mixed with an aqueous saturated sodium chloride solution and extracted with a considerable volume of tetrahydrofuran to remove excess of Nam and sodium trifluoromethanesulfonate. After multiple cycles of dissolution in methanol and removal of inorganic salts by filtration with subsequent evaporation of methanol, the anomerically pure crude β-NR^+^Cl^−^ was obtained in 77% yield (calculated as related to α/β-1,2,3,5-tetra-*O*-acetyl-D-ribofuranose) as an oily substance. This product was subsequently converted into a crystalline form, as a solvate with 0.9 molar equivalents of methanol. After several weeks of crystallization, pure β-NR^+^Cl^−^ was isolated in 34% yield (calculated based on α/β-1,2,3,5-tetra-*O*-acetyl-D-ribofuranose).

Fouquerel et al. [33] described the use of an anion exchange resin for the synthesis of NR^+^Cl^−^ from NR^+^OTf^−^. In this publication, Amberlite IRA400-Cl was used in methanol at 0 °C, resulting in quantitative yield of NR^+^Cl^−^ after 2–3 hours of reaction time, followed by methanol removal.

**Scheme 8:**
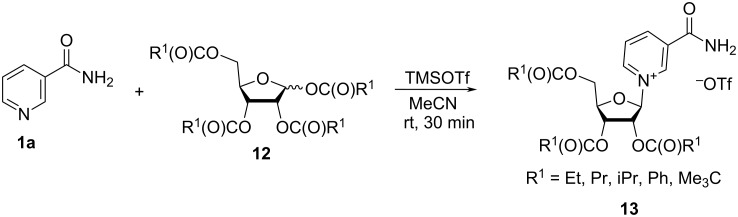
Synthesis of acylated NR^+^OTf^−^ by means of ion exchange with sodium chloride.

The patent publication of Szczepankiewicz et al. [34] describes the synthesis of a series of 1-(2′,3′,5′-tri-*O*-acyl-β-D-ribofuranosyl) nicotinamides **13** from corresponding 1,2,3,5-tri-*O*-acyl-α/β-D-ribofuranoses **12** (Scheme 8). In this patent, the authors used again a 3:1:5.5 ratio of Nam/tetraacyl riboside/TMSOTf to prepare the corresponding acylated NR^+^ triflates, that were then used for further transformations without purification. The authors do not report ratios of α- and β-anomers or the yields, except for the pivaloyl derivative (77%) that was purified via silica gel chromatography.

Mechanochemical methods (ball milling) of glycosylation of silylated nicotinamide **10a** with 1 equivalent of 1,2,3,5-tetra-*O*-acetyl-β-D-ribofuranose (**2a**) in the presence of 5 equivalents of TMSOTf are also reported [35]. The reagents were ball-milled at 25 s^−1^ for 30 min in a steel jar on a Retsch MM400 mill. The same procedure was used to react 1-*O*-acetyl-2,3,5-tri-*O*-benzoyl-D-β-ribofuranose (**2b**) with TMS-nicotinamide **10a** or 1,2,3,5-tetra-*O*-acetyl-D-β-ribofuranose (**2a**) with ethyl nicotinate (**14**). The glycosylation products were used without additional purification in a subsequent reduction step to yield the corresponding 1,4-dihydronicotinamide (nicotinate) ribosides.

A recent patent by Felczak [36] describes the synthesis of 2,3,5-tri-*O*-acetyl-NR^+^Cl^−^ **4a** in the reaction of 2,3,5-tri-*O*-acetyl-D-ribofuranosyl chloride (**3a**), prepared from 1,2,3,5-tetra-*O*-acetyl-β-D-ribofuranose (**2a**), with N-silylated Nam **10a**, followed by the removal of the acetyl groups in anhydrous methanol saturated with ammonia gas to yield NR^+^Cl^−^. The authors highlighted that an advantage of this approach was the improved solubility of the silylated derivative **10a**, thought to improve reaction rates with the chlorosugar **3a**, in non-polar solvents and thus thought to promote better β-stereoselectivity. Yet, compound **4a** was prepared in 28% yield and was contaminated with Nam. The removal of acetyl groups from **4a** gave NR^+^Cl^−^ in 38% yield as a mixture of β- and α-anomers, with β/α ratio being equal to 3:2.

**Scheme 9:**
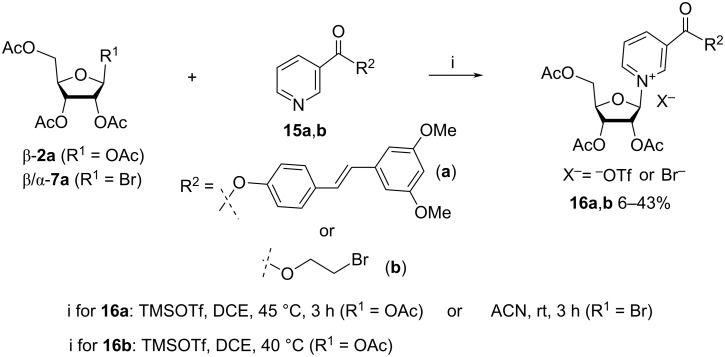
Synthesis of triacetylated derivatives of NAR^+^ by glycosylation of nicotinic acid esters with ribose tetraacetate or ribosyl-1-bromide triacetate.

The work of Migaud et al. [28] describes novel vitamin B3 conjugates, which were generated by glycosylation of nicotinate derivatives with 1,2,3,5-tetra-*O*-acetyl-β-D-ribofuranose (**2a**) or 2,3,5-tri-*O*-acetyl-α/β-D-ribofuranosyl bromide (**7a**, Scheme 9). For example, pterostilbene nicotinate **15a** was glycosylated with **2a** in the presence of TMSOTf in dichloroethane to give pterostilbene nicotinate riboside **16a** as a triflate salt in 18% isolated yield. However, the reaction of pterostilbene nicotinate with 2,3,5-tri-*O*-acetyl-α/β-D-ribofuranosyl bromide (**7a**) in acetonitrile afforded the desired β-anomeric pterostilbene nicotinate riboside bromide in 6% yield only. On both accounts, the lability of the pterostilbene ester bond is likely responsible for the extremely low isolated yields. The publication also mentions the glycosylation of 2-bromoethyl pyridine-3-carboxylate with 1,2,3,5-tetra-*O*-acetyl-β-D-ribofuranose to yield the corresponding 2-bromoethyl nicotinate riboside triacetate triflate **16b** in 43% yield.

**Scheme 10:**
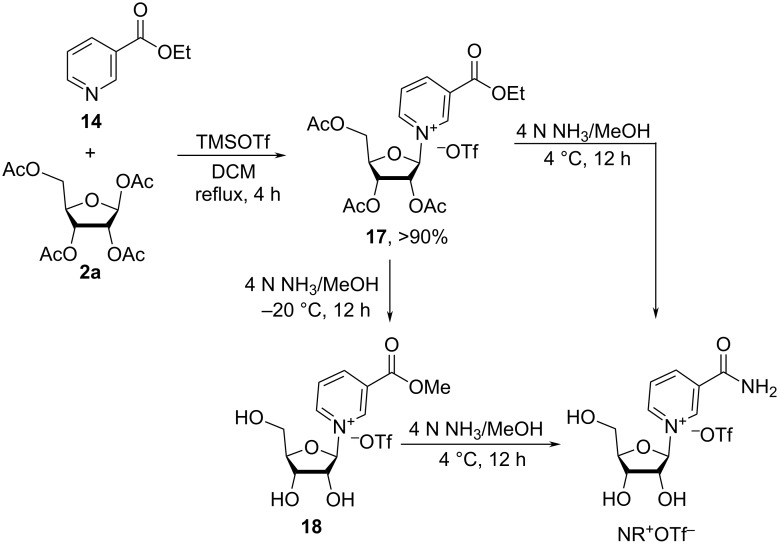
Synthesis of NR^+^OTf^−^ from the triflate salt of ethyl nicotinate-2,3,5-triacetyl-β-D-riboside in methanolic ammonia by simultaneous removal of the acetyl groups and transformation of ethyl nicotinate into nicotinamide riboside, as adapted from [37].

A new two-step one-pot procedure for the synthesis of β-NR^+^ was reported by Sauve et al. (Scheme 10) stemming from successively improved synthetic approaches [37–39]. The first step consisted in coupling of 1,2,3,5-tetra-*O*-acetyl-β-D-ribofuranose (**2a**) with ethyl nicotinate (**14**) in the presence of 1 equivalent of TMSOTf in dry dichloromethane resulting in the stereoselective formation of the corresponding β-isomer **17** of *O*-ethyl nicotinate riboside in high yield. It was speculated that less polar solvents, such as DCM, contribute to the formation of a less dissociated transition state, similar to **F** in Figure 3 above, which eventually leads to improved stereocontrol.

Treatment of 2,3,5-tri-*O*-acetyl-β-ethyl nicotinate riboside **17** with 4 N methanolic ammonia at 4 °C resulted in the simultaneous removal of the acetyl groups and the conversion of the ethyl nicotinate to the corresponding amide. A mechanistic study of this reaction revealed that it proceeded via the methyl nicotinate **18** which could be isolated when the transformation was carried out at −20 °C. These results indicated that methoxide is more reactive than ammonia in reacting with ester **17**. A very detailed synthetic procedure for the preparation of β-nicotinamide riboside triflate from ethyl nicotinate and 1,2,3,5-tetra-*O*-acetyl-D-β-ribofuranose is provided in this article [39]. The purification of the final NR^+^OTf^−^ was achieved by reversed phase column chromatography on a C18 column (activated with MeOH/water systems) using water as an eluent.

**Scheme 11:**
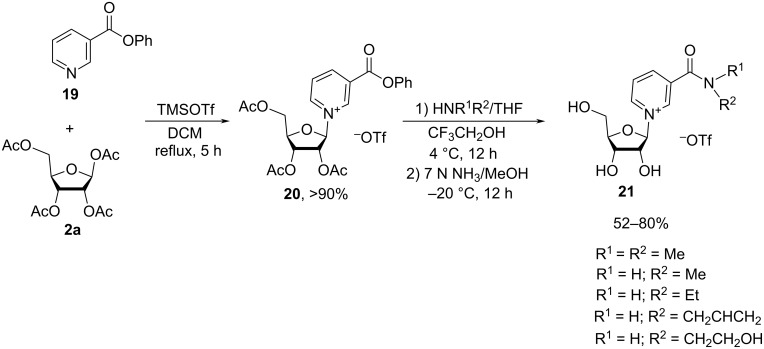
Reaction of 2,3,5-tri-*O*-acetyl-β-phenyl nicotinate riboside triflate salt with secondary and tertiary alkylamines followed by acetyl deprotection resulting in *N*-alkyl-substituted analogues of NR^+^OTf^−^, as adapted from [37].

The authors also attempted to use the newly discovered reaction for the synthesis of *N*-alkyl-nicotinamide ribosides by reaction of **17** with alkylamines in methanol at 4 °C. However, these amines promoted the decomposition of the nucleoside and cleavage of the glycosidic bond, as the major products were the corresponding *N*-alkyl-nicotinamides. A more reactive 2,3,5-tri-*O*-acetyl-β-phenyl nicotinate riboside **20** was therefore generated. To limit transesterification and favor amidation, trifluoroethanol was used as solvent instead of methanol, and as a result, amides **21** were prepared in good yields (Scheme 11).

In summary, glycosylation of silylated nicotinoyl derivatives with 1,2,3,5-tetra*-O*-acetyl-D-β-ribofuranose in the presence of trifluoromethanesulfonate proves to be the most efficient method to selectively produce in good yields the β-anomer scaffold of NR^+^ and NAR. Table 1 summarizes currently available data on the syntheses of nicotinamide riboside salts and acylated precursors thereof.

**Table 1:** Summary of the syntheses of triacylated nicotinamide (or ethyl nicotinate) riboside salts 2,3,5-triacyl-NR^+^Xˉ (or 2,3,5-triacyl-NAR^+^Xˉ) and nicotinamide riboside salts NR^+^Xˉ.

starting reagents		glycosylation reaction conditions: solvent/temp/time, Lewis acid	deacylation reaction conditions	product and yield, %		ref.
	
Nam or derivative or analogue	sugar			2,3,5-triacyl- NAR^+^X^−^	β-NR^+^X^−^	

**1a**	α/β-**3a**	MeCN/ 0 °C/42 h	NH_3_/MeOH, 0 °C, 18 h	α/β-**4a**, 62%	X = Cl, yield not given	[20]
**1a**	α/β-**3b**	MeCN/ 0 °C/36 h	NH_3_/MeOH, 0 °C, 18 h	α/β-**4b**, 40%	X = Cl, 73% (β/α ca. 4:1)	[20]
**1a**	α/β-**5**	MeCN/ rt/2 h	1 M NH_3_/MeOH, −15 °C, 4 days	**6** (β/α ca. 4:1), 91%	X = Cl, 34% (ca. 85% of β)	[22]
**1a**	α/β-**3a** (ca. 94% of β)	MeCN/ 70 °C/20 min	1.25 M HCl/MeOH, rt, 12 h	α/β-**4a**, 62% (β/α ca. 6:4)	X = Cl, yield not given	[25]
**1a**	α/β-**7b**	MeCN/ −5 °C/48 h	–	β-**8b**, 61%	–	[22]
**1a**	α/β-**7a**	SO_2_/−10 °C/ overnight	–	β-**8a**, 96%	–	[26]
**1a**	α/β-**7b**	SO_2_/−10 °C/ overnight	NH_3_/MeOH, −18 °C, 72 h	β-**8b**, 90%	X = Br, 55%	[26]
**1a**	α/β-**7a** (β/α ca. 1.5:1)	SO_2_/−10 °C/ overnight	1 M NH_3_/MeOH, −5 °C, 20 h	β-**8a**, 90% (β/α ca. 25:1)	X = Br, 80%	[27]
**1a**	α/β-**7a** (β/α ca. 1.5:1)	MeCN/ −15 °C	–	β-**8a**, 65%	–	[27]
**1a**	β-**2a**	MeCN/rt/1 h, TMSOTf	MeOH, rt, 30 min	α/β-**9a**, yield not given	X = OTf, 58% (β/α 87:13)	[29]
**10a**	β-**2a**	DCE/45 °C/2 h, TMSOTf	NH_3_/MeOH, −5 °C, 6 h	β-**9a**, 96%	X = OTf, 45%	[30]
**10a**	β-**2b**	DCE/45 °C/2 h, TMSOTf	NH_3_/MeOH, −5 °C, 48 h	β-**9b**, 93%		[30]
**1a**	α/β-**2a**	MeCN/rt/1 h, TMSOTf	1 M MeONa/MeOH, 3−5 °C, 40 min	α/β-**9a**, yield not given	X = Cl (ion exchange), 34% (MeOH solvate)	[32]
**14**	β-**2a**	DCE/45 °C/8 h, TMSOTf	5.5 M NH_3_/MeOH, 0 °C, 15−18 h	β-**17**, >90%	X = OTf, 85%	[39]

#### 2.2. Condensation of *N*-(2,4-dinitrophenyl)-3-carbamoylpyridinium salt with derivatives of D-ribofuranosylamine

**Scheme 12:**
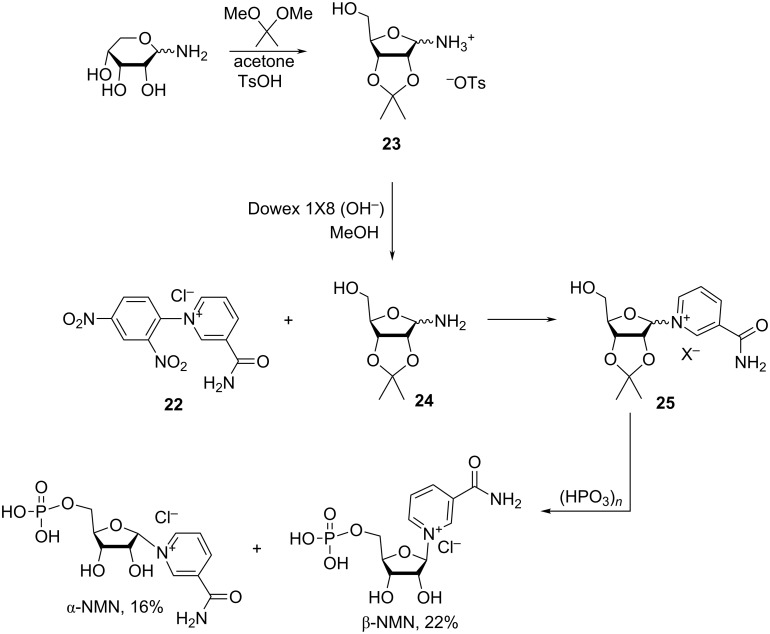
Synthesis of NMN based on the Zincke reaction of *N*-(2,4-dinitrophenyl)-3-carbamoylpyridinium chloride with 2′,3′-*O*-isopropylidene-D-ribofuranosylamine [17–18].

An alternative method to the preparation of nicotinamide riboside derivatives is based on the Zincke reaction of *N*-(2,4-dinitrophenyl)-3-carbamoylpyridinium chloride (**22**) with 2′,3′-*O*-isopropylidene-α/β-D-ribofuranosylamine (**24**, Scheme 12).

The synthesis is described in detail in the works of Jeck et al. [17–18], whereby 2′,3′-*O*-isopropylidene-α/β-D-ribofuranosylamine (**24**) was prepared from its *p*-toluenesulfonate **23** [40] by treatment of the latter with Dowex 1X8 resin (OH^−^ form) in methanol. The free amine (1.2 equivalents) was reacted with *N*-(2,4-dinitrophenyl)-3-carbamoylpyridinium chloride (**22**) overnight in methanol at room temperature. A crude mixture of α/β-*N*-(2,3-*O*-isopropylidene-D-ribofuranosyl)-3-carbamoylpyridinium salt **25** was isolated following the filtration of 2,4-dinitroaniline, the removal of methanol, the trituration with ether, and finally the treatment with Dowex 1X8 resin (HCO_3_^−^ form). The mixture was then phosphorylated with metaphosphoric acid to produce a mixture of α/β-NMN. The individual anomers were separated by column chromatography on a Dowex 50 column (H^+^ form). β-NMN^+^Cl^−^ was obtained in 22% yield, and α-NMN^+^Clˉ (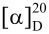 +59°) was obtained in 16% yield, with the yields being reported based on compound **24**.

The relatively low yield of β-NMN^+^Cl (low β-stereoselectivity) in the above described synthesis may be partially explained by the fast mutarotation of 2′,3′-*O*-isopropylidene-D-ribofuranosylamine and its tosylate. This mutarotation is solvent-dependent. For example, according to ^1^H NMR data presented in the work of Cusack et al. [40], compound **23** exists essentially as the pure β-anomer in chloroform whereas in dimethyl sulfoxide and water solutions, mixtures of α- and β-anomers are observed, with α/β ratio being 1:1.7 and 1:1.5, respectively. Moreover, mutarotation of the tosylate **23** is very fast in aqueous basic solution, being almost instantaneous in aqueous 2 M Na_2_CO_3_ as emphasized by the authors [40].

Atkinson et al. [23] showed that the reaction between a benzoylated derivative of D-ribofuranosylamine such as 2,3,5-tri-*O*-benzoyl-D-ribofuranosylamine and *N*-(2,4-dinitrophenyl)-3-carbamoylpyridinium chloride (**22**) yielded almost exclusively the α-anomer of NR^+^Cl^−^ (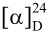 +46°). This was measured after de-esterification of the tribenzoate intermediate (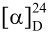 +25°) using methanol saturated with dry ammonia at 2 °C for 2 days (content of β-anomer was <3%, as determined by enzymatic analyses). This result may be explained by the fact that 2,3,5-tri-*O*-benzoyl-D-ribofuranosylamine – although prepared by reduction of anomerically pure 2,3,5-tri-*O*-benzoyl-β-D-ribofuranosylazide – undergoes rapid mutarotation resulting in an α/β anomeric mixture as demonstrated by Baddiley et al. [41].

**Scheme 13:**
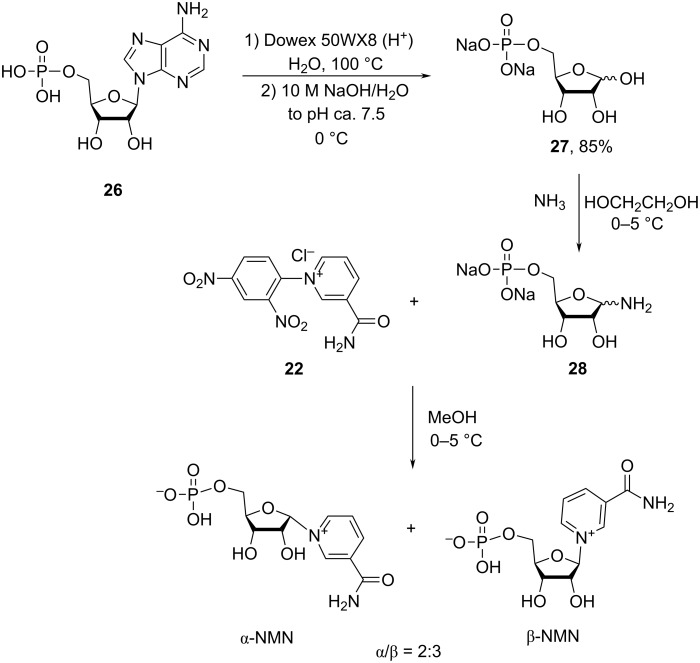
Synthesis of NMN based on the Zincke reaction of *N*-(2,4-dinitrophenyl)-3-carbamoylpyridinium chloride with 1-amino-ribose 5-phosphate, as adapted from [42].

Subsequently, Walt et al. [42] described the synthesis of NMN based on the above methodology but starting from the 5-phosphoribose obtained by acid hydrolysis of adenosine monophosphate (AMP, **26**, Scheme 13). In the first step, AMP was hydrolyzed under action of a Dowex resin in an aqueous medium at 100 °C to give 5-phosphoribose isolated as the disodium salt **27** in 85% yield with a purity of 88%. Subsequent amminolysis was performed by bubbling anhydrous ammonia gas into a suspension of anhydrous 5-phosphoribose in anhydrous ethylene glycol. After reaction completion, ammonia was removed under reduced pressure and the 5-phosphorylated aminoribose **28** reacted with *N*-(2,4-dinitrophenyl)-3-carbamoylpyridinium chloride (**22**) in anhydrous methanol at 0–5 °C for 14 h to afford a 3:2 mixture of β- and α-anomers of NMN (as determined by ^1^H NMR) in good to moderate yields (24–60% based on 5-phosphoribose **27**) depending on the reaction scale, with lower yield being achieved at the larger scale due to difficulties in removing ammonia. β-NMN was not isolated but used as to prepare NAD^+^ enzymatically.

While the condensation of *N*-(2,4-dinitrophenyl)-3-carbamoylpyridinium salt with derivatives of D-ribofuranosylamine gives mixtures of α- and β-anomers of NR^+^ derivatives which require fine-tuning and delicate purifications, it also provides direct access to the non-acylated forms of NR^+^, and NMN, or even mainly α-NR^+^.

#### 2.3. Enzymatic synthesis of NR

Nicotinamide riboside may be prepared enzymatically from NAD^+^ and NMN by several procedures. Kaplan and Stolzenbach [43] proposed an enzymatic cleavage of NAD^+^ to NMN by using snake venom phosphodiesterase (prepared from *Crotalus adamanteus* venom) and subsequent transformation of NMN to NR^+^ under catalysis with a prostatic monoesterase. Kasasarov and Moat [44] used a crude enzyme preparation from *Proteus vulgaris* OX-19 to prepare NR^+^ from NAD^+^, with the process being conducted at 37 °C. This set of conditions made it possible to stop enzymatic reactions at the stage of the nicotinamide riboside formation (i. conversion of NAD^+^ to NMN catalyzed by pyrophosphatase and ii. conversion of NMN to NR^+^ catalyzed by 5′-nucleotidase) because enzymes catalyzing further degradation to Nam and nicotinic acid (nucleosidase and amidase, respectively) were inactivated at that temperature. An article of Saunders et al. [45] describes the preparation of NR^+^ by treating NMN with a commercially available 5′-nucleotidase. The product was purified by ion exchange chromatography on AG 1-X8 column using water as eluent.

The enzymatic synthesis of NR^+^ from α-D-ribose-1-phosphate and Nam by catalysis with purine nucleoside phosphorylase and sucrose phosphorylase is described in a US patent application by Velasquez et al. [46]. In the NMR experiments, the authors demonstrated the preparation of NR^+^ but did not isolate pure NR^+^.

### 3. Chemical modification and properties of NR

NR^+^ and its derivatives contain several chemical functional groups which present different reactivity of significance when seeking to introduce structural and functional modifications. The most common modifications are conducted on the C5′-hydroxy group, yielding conjugates of NR^+^. Modifications which include protection of 2′- and 3′-hydroxy goups and reduction of the pyridinium core are used when direct modifications at the 5′-position are not possible due to the cleavage of the glycosidic bond under the required conditions, or are troublesome due to the low reactivity of the 5′-OH. Modifications of the carboxyl functionality have also been explored to generate amide and ester derivatives of NR^+^ and nicotinic acid riboside (NAR), respectively.

#### 3.1 Stability of NR^+^ to Brønsted acids and bases

Unlike Nam and nicotinic acid (NA) which are chemically stable, NR^+^, NMN and NAD^+^ are readily hydrolyzed to Nam, with their rapid decomposition particularly promoted by heat and basic aqueous solutions (pH ≥ 8). Under non-buffered conditions, the hydrolysis of the glycosidic bond leads to the release of Nam, which possesses a p*K*_a_ at 3.35 [47]. The release of Nam results in the in situ generation of pyridinium hydroxide, resulting in further NR^+^ hydrolysis. This unfavorable reactivity of the C-1′ position was briefly addressed in the earlier sections of this review, as evidenced by the needs of anhydrous conditions and low temperatures in the deprotection steps to generate NR^+^X^−^ from the triester precursors.

Because of high biological relevance of NAD(P)^+^ and NADH(P), many studies on the stability of these dinucleotides to pH and temperature have been undertaken [48–55]. Generally, NR^+^ and NAD^+^ are most stable at lower pH of 5.5–6.0 whereas reduced forms are stable in slightly basic media (pH 9.0–9.2) [56]. When pH values are beyond these ranges, compounds may undergo fast decomposition, the rate of which increases with increasing temperature.

The profile of the hydrolysis of NAD^+^ as a function of pH is complex [49]. As such, the hydrolysis is pH independent at pH below 6.5 and above 12.5. However, in the pH range from 8.5 to 11.0 the hydrolysis reaction shows first order kinetics in OH^−^ (i.e., the hydrolysis rate constant is linearly dependent on pH with a slope of unity). It was also shown that different hydrolysis products were formed depending on the pH of the hydrolytic medium [49]. When hydrolysis was performed in the presence of a rather dilute 0.1–1.0 N KOH aqueous solution, Nam was identified as the main product of the hydrolytic cleavage. 2-Hydroxy-3-pyridinecarboxaldehyde (2-HPC) became the predominant product under more concentrated alkaline conditions (5 N KOH) as a result of a pyridinium ring opening–closure process [57]. It was also shown that the ionization of a diol fragment of NAD^+^ under alkaline conditions plays a very important role in the dissociative cleavage of the glycosyl bond of the “northern” ribosyl. NR^+^ derivatives and isopropylidene protected NR^+^ is somewhat more stable to alkaline hydrolysis [49]. In the case of isopro-NR^+^, the hydrolysis involves an addition of a hydroxide ion to the pyridine core resulting in formation of 2-HPC [49].

It is worth mentioning that NR^+^ derivatives are not stable in pyridine even at room temperature suffering transformations into the corresponding quaternary pyridine ribofuranoside [20].

These observations highlight the need for special appreciations for the chemical stability of the glycosidic bond in NR^+^ when conducting chemical reactions and choosing appropriate work-up procedures for NR^+^/NRH derivatives, as this class of nucleoside greatly differs from the canonical purine and pyrimidine nucleosides. For instance, the glycosyl bond of NR^+^ was shown to be very susceptible to cleavage in methanolic solutions of ammonia [39]. As such, to get the corresponding deprotected NR^+^ salt in good yields and free of admixtures, removal of acetate groups from 3-(carbamoyl)-1-(2,3,5-tri-*O*-acetyl-β-D-ribofuranosyl)pyridinium salt (NR^+^ triacetate) should be conducted under specifically controlled conditions (e.g., using ammonia in anhydrous methanol at −3 to −5 °C as recommended by Lee et al. [27]), and with subsequent – after the completion of deacetylation – evaporating excessive ammonia at temperatures also below 0 °C [25,39]. Moreover, because of the limitations associated with different stability to different pH ranges, the reduction of NR^+^ to NRH must be performed in slightly basic media, such as saturated sodium bicarbonate solution [19,31].

#### 3.2. Protection of 2′,3′-hydroxy groups

**Scheme 14:**
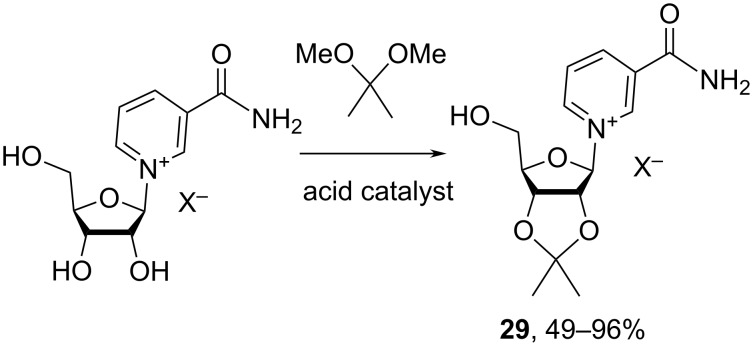
Efficacious protection of 2′,3′-hydroxy groups of NR^+^X^−^.

As it is the case for most synthetic efforts associated with nucleosides’ syntheses the protection of the 2′,3′-hydroxy groups of the furanosides is required when these interfere with planned chemical modifications of NR^+^X^−^ on the 5′-C-hydroxy moiety. The most common approaches convert the *cis*-2′,3′-hydroxy groups of NR^+^X^−^ to an acetonide (i.e., 2′,3′-*O*-isopropylidene nicotinamide riboside **29**; isopro-NR) in a reaction with 2,2-dimethoxypropane in the presence of an acid catalyst (Scheme 14).

Conditions which use *p*-toluenesulfonic acid in acetone [58] produced acetonide **29** in 49% yield after column chromatography. Conditions which employed concentrated sulfuric acid in acetonitrile [59–60] yielded the acetonide in 76–96% yield. Large excess of 2,2-dimethoxypropane is usually used (8–10 molar equivalents relative to NR^+^). It should be noted that possible anion exchanges in the isolated product were never addressed. Jarman et al. [22] also described the protection of 3-carbamoyl-4-methyl-1-(D-ribofuranosyl)pyridinium chloride (4-Me-NR^+^Cl^−^) in dry DMF in the presence of a 6 M HCl dioxane solution (almost equimolar amount relative to 4-Me-NR^+^Cl^−^) to give the corresponding acetonide in 88% yield.

**Scheme 15:**
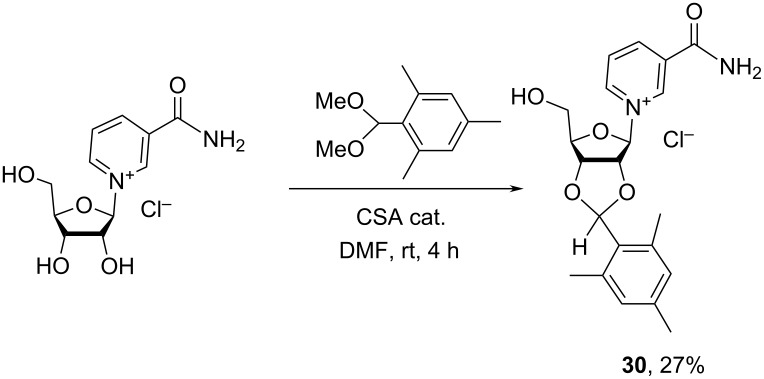
Protection of the 2′,3′-hydroxy groups of NR^+^Cl^–^ with a mesitylmethylene acetal group.

The use of dimethyl acetal of mesitylaldehyde in the presence of catalytic amounts of 10-camphorsulfonic acid in dry DMF was reported [58] but the isolated yield for the corresponding protected derivative **30** was low (Scheme 15). Yet, this particular protecting group is easily removed under mild acidic conditions, such as diluted HCl in organic solvents (THF/MeOH) or 90% aqueous trifluoroacetic acid or hydrogenolysis.

#### 3.3. Reduction of the pyridinium core of NR^+^ and its derivatives

**Figure 5:**
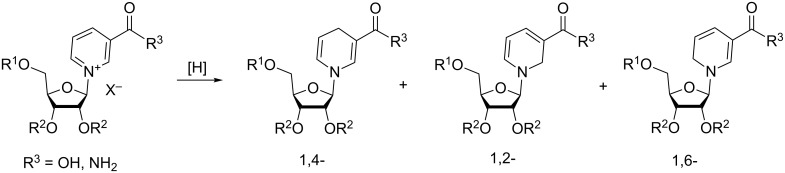
Reduction of derivatives of NR^+^Xˉ into corresponding 1,2-; 1,4-; 1,6-NRH derivatives.

Chemical reduction of N-substituted pyridinium salts may result in three isomeric products: 1,2-, 1,4-, and 1,6-dihydropyridines (DHP) as illustrated in Figure 5 for the corresponding dihydro-1-β-D-ribofuranosyl-3-pyridinecarboxamides.

The reduction of pyridinium salts to dihydropyridines has been well studied and extensively reviewed [61–64]. It was demonstrated that the outcome of the reduction reaction is dependent on many factors, such as the electron withdrawing/releasing properties and locations of the substituents on the pyridinium core. They also depend on the reducing reagent, the solvent used for the reaction and the reaction temperature.

Sodium borohydride (NaBH_4_) and sodium dithionite (Na_2_S_2_O_4_) are the most studied reducing agents for the reduction of N*-*substituted pyridinium derivatives. Unlike Na_2_S_2_O_4_ which reduces NAD^+^ regioselectively to 1,4-dihydronicotinamide adenine dinucleotide (NADH), NaBH_4_ reduces NAD^+^ to a mixture of the 1,2-, 1,4-, and 1,6-NADH isomers [65–66]. Recent attempts to reduce substituted pyridinium salts using less powerful and more selective sodium cyanoborohydride also failed to produce 1,4-DHP derivatives in good yields [56]. As a general observation, one may note that dithionite reduction of pyridinium salts, especially those carrying electron-withdrawing substituents in the 5 and 3 positions of the pyridinium core, offers mainly or even exclusively the 1,4-DHP products. NaBH_4_ reduction does not demonstrate such a selectivity [67].

**Figure 6:**
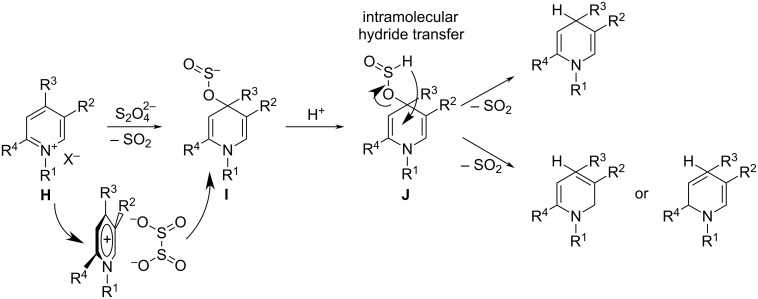
Mechanism of the reduction of the pyridinium core with dithionite as adapted from [67].

Mechanistic studies of Na_2_S_2_O_4_ reduction of pyridinium salts, including NR^+^ derivatives, suggest that this reaction proceeds through the formation of sulfinic acid adducts (1,4-dihydro-4-pyridine sulfinates) **I**. This adduct is generated from the completely regioselective attack of the dithionite ion on the 4 position of the pyridinium core (see Figure 6). This regioselectivity is explained by the particular mutual orientation of the pyridinium cation and the dithionite anion and by the match of interatomic distances between the two negatively charged oxygen atoms in dithionite and the positively charged pyridinium nitrogen and the electrophilic C4 atom. Rearrangement of the protonated intermediate **J** with elimination of SO_2_ usually affords the 1,4-dihydropyridines with a very low admixture of other isomers. The 1,2- or 1,6-dihydropyridines may become predominant products depending on a particular combination of substituents R^1^, R^2^, R^3^, and R^4^ [67–69]. For instance, in the case of starting substrate **H**, in which R^1^, R^3^, R^4^ = Me and R^2^ = CN, the reduced product contained 70% of 1,2-dihydropyridine and 30% of 1,4-dihydropyridine as established by NMR spectral analysis. Reduction of *N*^1^-methyl-3-carbamoylpyridinium iodide, R^1^ = Me, R^2^ = C(O)NH_2_, gave a mixture composed of 82% of 1,2- and 18% of 1,6-isomers. At the same time, reduction of *N*^1^-benzyl-3-carbamoylpyridinium bromide provided a 95:5 mixture of 1,4-/1,6-dihydropyridines while *N*^1^-benzyl-3-carbamoyl-6-methylpyridinium bromide was reduced to yield only the 1,4-product. Therefore, according to Carelli et al. [67], the different outcomes of the reduction of variously substituted pyridinium salts depend not on the selectivity of dithionite attack – which regioselectively occurs on the 4-position of the pyridinium core to give intermediate **J** – but rather on the respective thermodynamic stability of the dihydro derivatives.

**Scheme 16:**
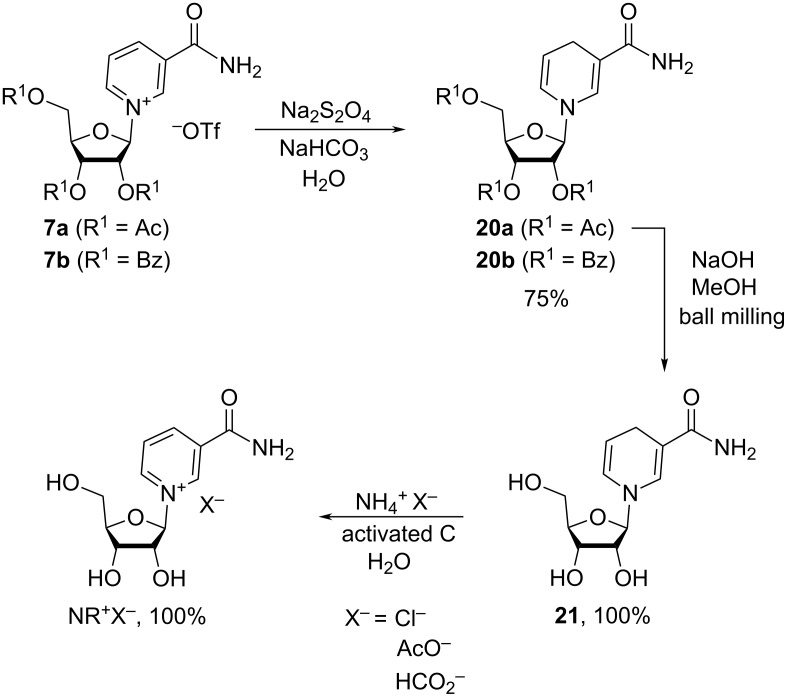
Reduction of triacylated NR^+^OTf^–^ derivatives by sodium dithionite followed by complete removal of acyl groups and re-oxidation on charcoal to give pharmaceutically acceptable salts NR^+^X^−^.

Application of the dithionite reduction to the synthesis of 2,3,5-tri-*O*-acetyl- and 2,3,5-tri-*O*-benzoyl-D-β-1,4-dihydronicotinamide ribosides [19,31,35], their tripropionate, tri-*n*-butyrate, triisobutyrate, triethyl carbonate, tripivalate, 5-*O*-myristolate analogues [34], 2′,3′-*O*-isopropylidene-1,4-dihydronicotinamide riboside [60], and 1,4-dihydronicotinate ribosides [70]. Typically, the reduction with Na_2_S_2_O_4_ is performed under mild basic conditions (e.g., in a medium of aqueous sodium bicarbonate or potassium phosphate dibasic) due to the instability of the reduced products in acidic media. According to a patent publication [35], the triacetyl and tribenzoyl dihydronicotinamide ribosides were synthesized in the yields of ca. 75% (Scheme 16). Exhaustive deprotection of the acetyl groups from **20a** with sodium hydroxide in the presence of methanol under ball milling conditions (30 min at 25 Hz) resulted in the corresponding 1,4-dihydronicotinamide riboside NRH **21** isolated in quantitative yields. Alternatively, this deprotection procedure was conducted with 1 M sodium methoxide in MeOH over a longer period of time [58].

1,4-Dihydronicotinamide riboside, NRH, may be subsequently oxidized into NR^+^ salts using activated charcoal [31], salts for which a pharmaceutically acceptable counter anion X^−^ can be introduced when using an aqueous medium containing the corresponding ammonium salt NH_4_X. The oxidation may also be achieved with hexachloroacetone [28,71] or cobalt(II) acetate in the presence of hydrogen peroxide followed by removal of the cobalt cations with QuadraSil AP resin [60]. The nature of the counter anion vary as a function of the conditions applied.

**Figure 7:**
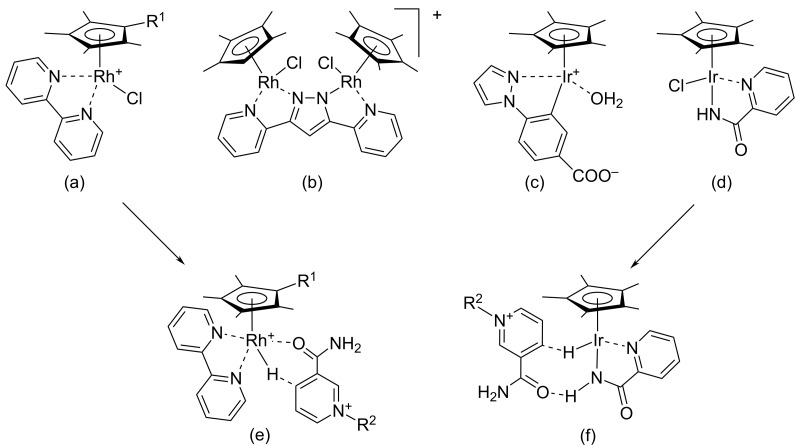
Structural formulas of iridium and rhodium catalysts (a)–(d) for regeneration of NAD(P)H from NAD(P)^+^ and transition states (e) and (f).

Reduction of *N*^1^-substituted 3-nicotinamide salts to the corresponding 1,4-dihydropyridine derivatives may be also accomplished by using catalysis with organometallic complexes [72–79] or platinum nanoparticles [80]. This recently developed approach stems from an independent field of research, which focuses on the regeneration of NAD(P)H from NAD(P)^+^ [81–82]. Many of these complexes contain rhodium or iridium coordinated with fully (methyl)substituted cyclopentadienyl ligands and bis-coordinating diamino ligands as shown in Figure 7. The reducing agent may be a formate anion as a source of hydride, triethanolamine as an electron donor in the case of photoactivated reduction, or hydrogen gas under atmospheric pressure. Some of these complexes were used in the successful electrochemical regeneration of NAD(P)H [83].

According to the proposed mechanism of the complex-catalyzed reduction [75], reaction of a complex with, for example, formate generates a hydride metal complex. The amide functionality in the 3-nicotinamide core plays a crucial role in the subsequent reduction because it coordinates with the metal center of the hydride complex, resulting in a favorable transition state which ensures the transfer of the hydride on the 4-C position of the pyridinium ring (see Figure 7).

A comprehensive review on the chemistry of homogeneous and heterogeneous catalytic, electrochemical, photocatalytic, and immobilized catalysts-based regeneration of NAD(P)H was published by Wang et al. [84].

#### 3.4. Modifications on the 5′-position of unprotected and partially protected nicotinoyl ribosides

**Figure 8:**
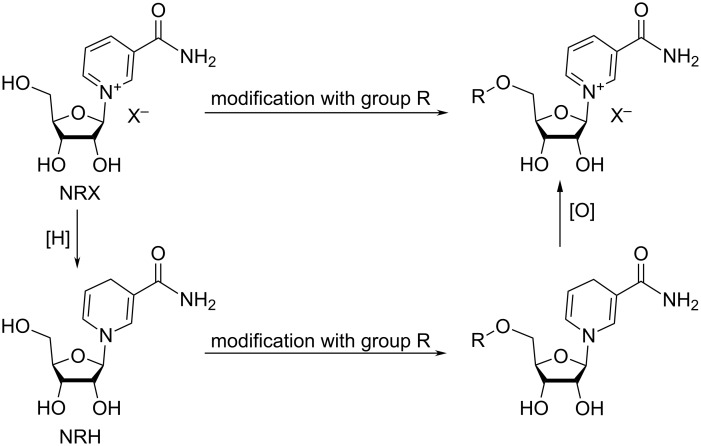
Two approaches to synthesis of 5′-derivatives of NR^+^.

Currently, there exist two main approaches for the synthesis of 5′-modified derivatives of NR^+^ and they are schematically illustrated in Figure 8. The first direct approach consists in the reaction of the unprotected or partially protected NR^+^ salts with electrophilic reagents. The second approach requires the initial conversion of NR^+^ to NRH. The later allows increased reactivity of the C5′-hydroxy group under conditions which otherwise lead to the rapid decomposition of NR^+^. Furthermore, the C5′-OH in NR^+^ shows reduced nucleophilic properties, probably as a consequence of interactions with the heteroaromatic moiety, as described by Tang et al. for 2′-deoxycytidine [85]. Therefore, modifications at the C5′-position of the NRH parents followed by the re-oxidation of the conjugated reduced forms allow for an easier access to the desired 5′-modified NR^+^ products.

**Scheme 17:**
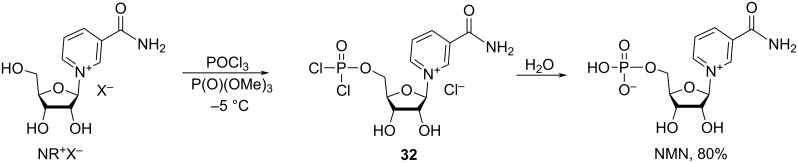
Synthesis of NMN starting from NR^+^ salt.

The C-5′-position is most commonly modified to generate the phosphorylated derivative of NR^+^, nicotinamide mononucleotide, NMN. The phosphorylation is achieved by reacting NR^+^ in its salt form with phosphorus oxychloride in trimethyl phosphate resulting, after hydrolysis, in NMN (Scheme 17). Lee et al. [27] carefully studied this reaction and found that the phosphorylation step should be conducted under strictly controlled temperature conditions in the range of −5 to 0 °C to prevent the undesired 5′-chlorination which occurs at higher temperatures, and to avoid slow reaction rates at lower temperatures. Lee et al. [27] also showed that the reaction of β-NR^+^Br^−^ and 4 equivalents of POCl_3_ in P(O)(OMe)_3_ conducted at this temperature range for 7 h provided – after subsequent hydrolysis of the chloride intermediate **32** – β-NMN in a yield of at least 80% with more than 97% purity achievable after column chromatography on Dowex 50WX8 resin.

According to the patent publication of Livingston et al. [60], the chloridate **32** may be reacted not only with water but also with alcohols, such as isopropanol, ethanol, propanol, methanol, butanol, to result in the mono-substitution of a chlorine atom in **32** with corresponding alkoxy group and – after quenching with water – to give NMN analogues with one alkoxy group at the phosphorus atom. The resulting compounds were purified by column chromatography on an aminopropyl-modified silica gel Quadrasil AP column to yield the pure NMN analogues in low to moderate yields (8–40%).

**Scheme 18:**
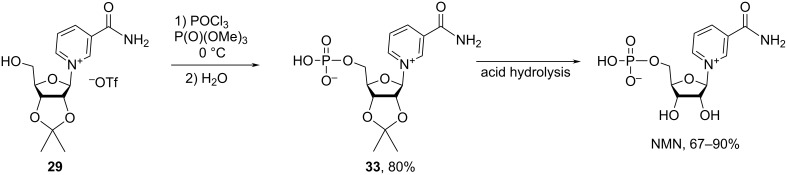
Efficient synthesis of NMN by phosphorylation of 2′,3′-*O*-isopropylidene-NR^+^ triflate followed by removal of acetonide protection.

The patent publication of Sauve et al. [59] divulges phosphorylation of isopro-NR^+^ **29** (as triflate salt) with phosphorus oxychloride in trimethyl phosphate at 0 °C for 48 h to give isopro-NMN **33** in 80% yield (Scheme 18) after silica gel column chromatography using a 6:4 DCM/MeOH mixture as an eluent. Subsequent deprotection of the NMN acetonide **33** using trifluoroacetic acid in DCM/water mixture or HCl in methanol was followed by C18 reverse phase chromatography to afford NMN in 90% and 67%, respectively.

**Scheme 19:**
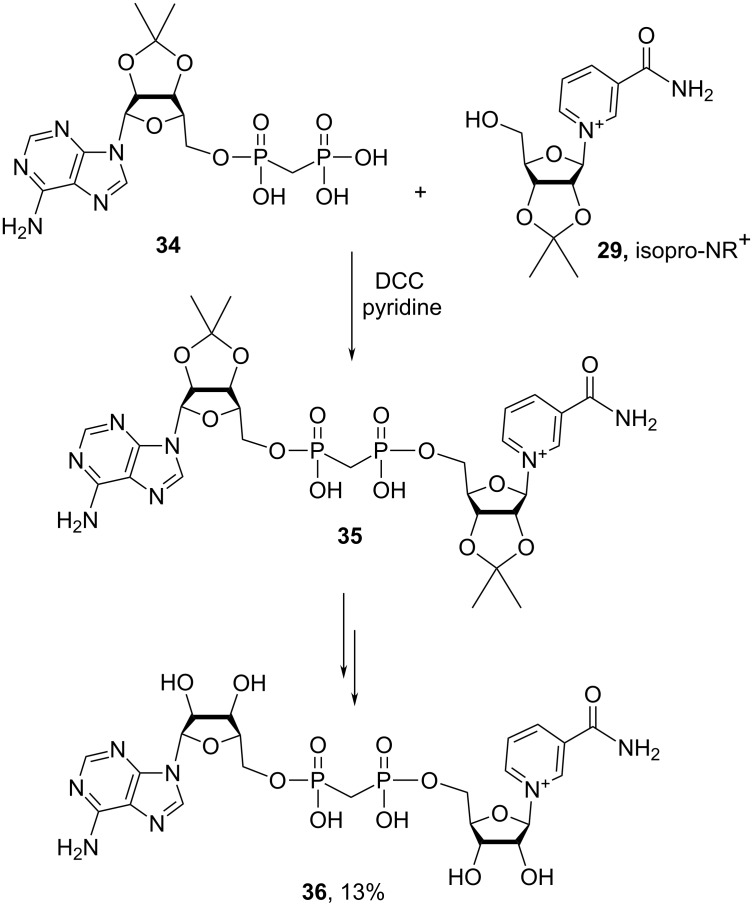
Synthesis of a bisphosphonate analogue of β-NAD^+^ based on DCC-induced conjugation of 2′,3′-*O*-isopropylidene-NR^+^ with adenosine bisphosphonate derivative.

Meyer and Hilz [86] reported the synthesis of the bisphosphonate analogue of β-NAD^+^ **36** using a synthetic sequence, the key reaction of which was a coupling of bisphosphonate **34** with isopropylidene-protected NR^+^ **29** under the Steglich esterification conditions in the presence of tri-*n*-octylamine, pyridine and DCC in DMF medium at 60 °C for 18 h (Scheme 19). Compound **36** was isolated in pure form by means of purification on a Dowex formate column and characterized by ^1^H NMR spectroscopy. The low isolated yield of the final β-NAD^+^ bisphosphonate derivative (13%) may be explained by the fact that isopro-NR^+^ used was a mixture of β/α anomers with respective ratio of 3:2 and that the coupling conditions used pyridine as solvent.

**Scheme 20:**
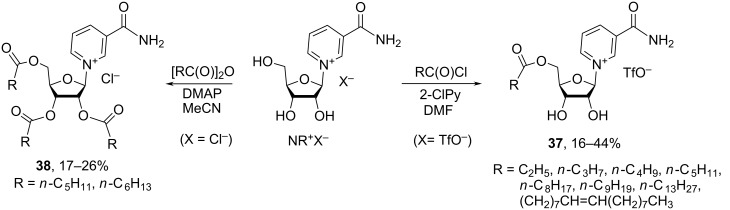
Synthesis of 5′-acyl and 2′,3′,5′-triacyl derivatives of NR^+^.

The patent publication of Szczepankiewicz et al. [34] describes the preparation of 5′-alkanoate and 2′,3′,5′-alkanoate derivatives of NR^+^ synthesized by the direct acylation of NR^+^Cl^−^ or NR^+^OTf^−^ with the corresponding acid anhydrides or acid chlorides (Scheme 20). Thus, the reaction of NR^+^OTf^−^ with alkanoyl chlorides, such as propionyl chloride, *n*-butyryl chloride, *n*-pentanoyl chloride, *n*-hexanoyl chloride, *n*-decanoyl chloride, *n*-tetradecanoyl chloride, oleoyl chloride, nonanoyl chloride, used in 7.3 equivalent excess to NR^+^OTf^−^, was carried out in DMF in the presence of 2-chloropyridine at room temperature for 30 min to yield the 5′-alkanoates **37** in low to moderate yields (16–44%) after HPLC reversed phase purification. At the same time, exhaustive acylation of NR^+^Cl^−^ was achieved with *n*-pentanoic anhydride or *n*-hexanoic anhydride, used in 2.7 or 4.5 molar ratio excess to NR^+^Cl^−^, when conducted in acetonitrile in the presence of DMAP for 18 h. After purification by silica gel chromatography, the 2′,3′,5′-tripentanoate and trihexanoate **38** were obtained in low yields of 17% and 26%, respectively.

The authors of the above patent also described exhaustive acylation of NR^+^ salts when using ethyl chloroformate. This yielded nicotinamide riboside triethylcarbonate but the authors were not able to completely purify the product. It should also be noted that all the described compounds were characterized only by mass spectrometry and the patent publication does not contain the NMR spectroscopic data to confirm the products’ identity.

**Figure 9:**
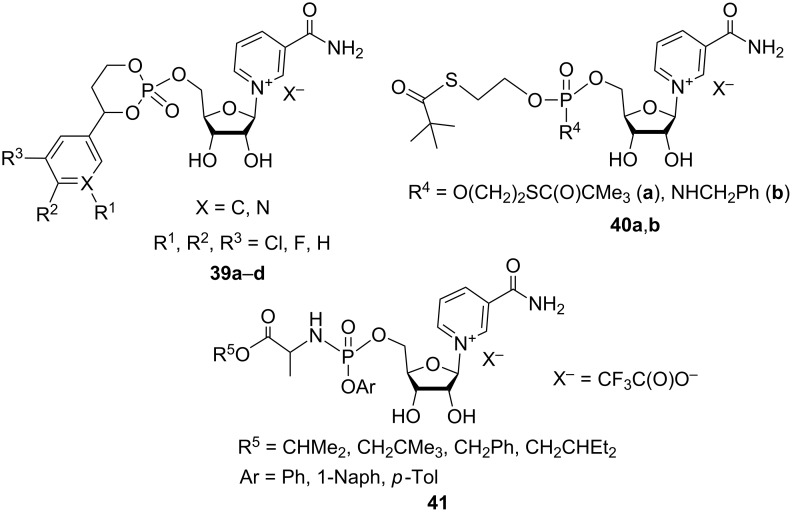
Structural formulas of NMN analogues **39**–**41**.

The patent publication of Livingston et al. [60] deals with the synthesis of NMN analogues **39**–**41** (Figure 9). These compounds were developed with the view of generating masked versions of NMN with pharmacological function in order to manipulate the NAD^+^ pathway in mammalian cells and tissues.

**Scheme 21:**
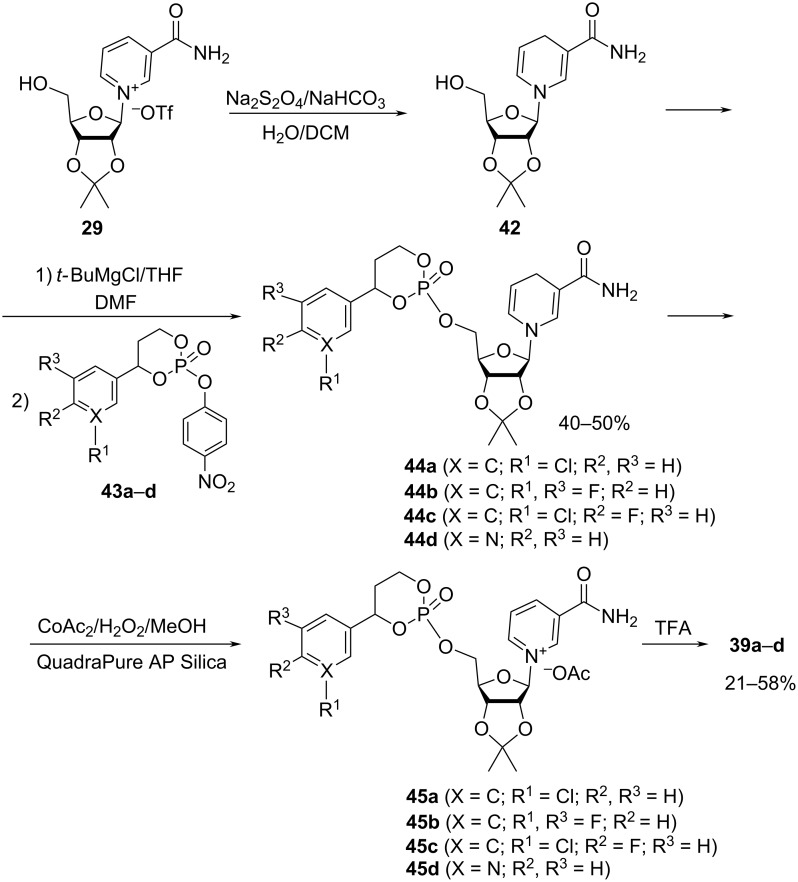
Synthesis of 5′-phosphorylated derivatives of NR^+^ using a “reduction–modification–oxidation” approach.

The key step in the synthesis of the NMN analogues **39**–**41** is the activation of the 5′-hydroxy group of the NRH acetonide **42** with a Grignard reagent, to produce the corresponding magnesium alkoxide. As illustrated by the synthesis of the compounds **44** (Scheme 21), subsequent phosphorylation of the alkoxide with electrophilic phosphate triesters yielded the phosphate esters **44** in ca. 40–50% yield, after purification by flash chromatography on silica gel (0–10% methanol in dichloromethane as an eluent).

These products were then oxidized using cobalt acetate in the presence of hydrogen peroxide. This approach required the subsequent removal of metal ions by Quadrasil resin. The oxidized acetonides **45** were subsequently deprotected using the mixture of 90% aqueous TFA/DCM and the resulting crude NMN analogues **39** were purified by silica gel column chromatography (10% MeOH in DCM as an eluent) resulting in the isolation of NMN derivatives achieved in overall isolated yields of 21–58% for the combined oxidation and deprotection steps.

**Scheme 22:**
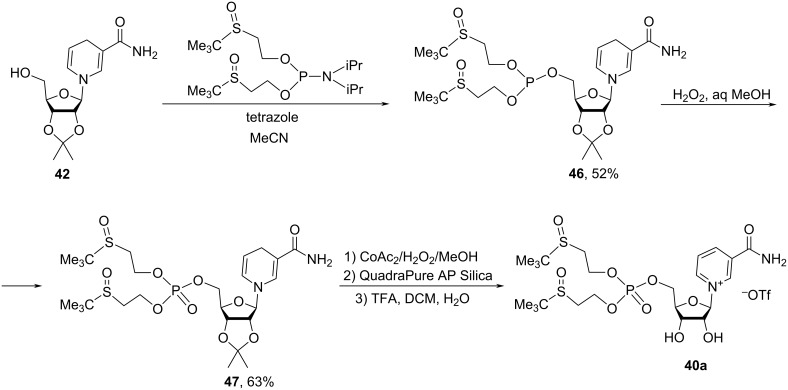
Synthesis of 5′-phosphorylated derivatives of NR^+^ using a “reduction–modification–reoxidation” approach.

Compound **40b** was prepared by an analogous procedure, while bis-*S*-pivaloyl analogue **40a** was synthesized by first reacting compound **42** with a phosphoramidite in the presence of tetrazole in ACN with the subsequent oxidation of the intermediate phosphite by hydrogen peroxide to yield the crude phosphate **47**. The latter was purified by silica gel column chromatography using a gradient eluent system from DCM to 3% MeOH/DCM (Scheme 22). Subsequent oxidation of the dihydropyridine core with cobalt acetate and removal of the acetonide in a trifluoroacetic acid in DCM/water mixture gave the bis-S-pivaloyl NMN analogue **40a**.

Finally, compounds **41** were synthesized following the same approach as that used in the preparation of the analogues **39a**–**d**, either using 4-nitrophenoxy phosphates (4-O_2_NC_6_H_4_)P(O)(OAr)(NR′) or chlorophosphates ClP(O)(OAr)(NR′) as phosphorylating agents.

**Figure 10:**
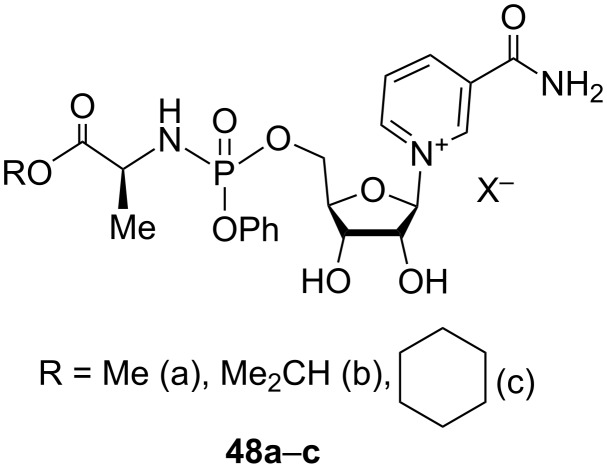
Structural formulas of 5′-phosphorylated derivatives of NR^+^.

**Scheme 23:**
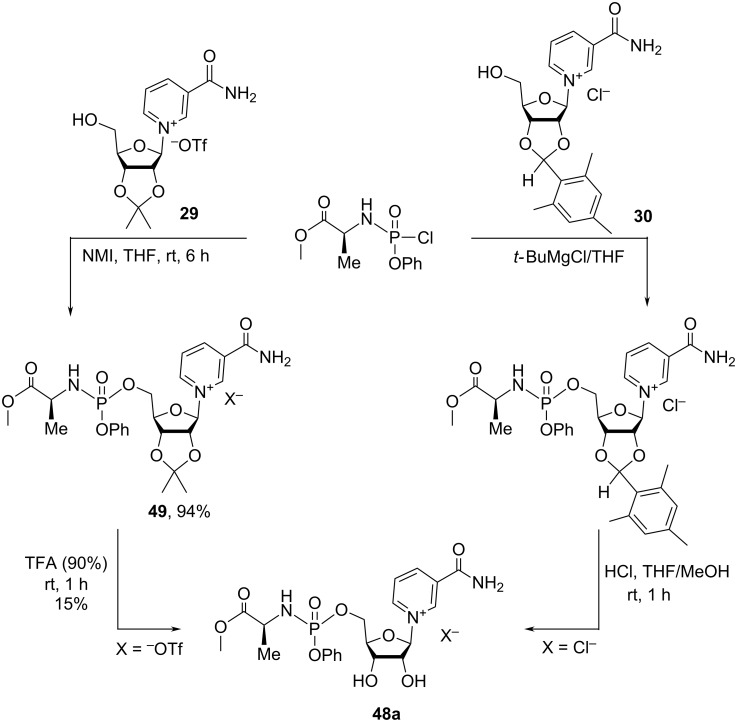
Synthesis of 5′-phosphorylated derivatives of NR^+^ using a direct NR^+^ phosphorylation approach.

The patent publication of Kluge et al. [58] also relates the synthesis of NMN analogues with general formula **48a**–**c** (Figure 10) with minor structural differences as compared to the derivatives **41** (e.g., different combination of R and Ph substituents in these molecules). In an initial approach to prepare compounds **48a**–**c**, the authors used the direct phosphorylation of the 2′,3′-*O*-isopropylidene nicotinamide riboside triflate, with a substituted phosphorus acid chloride in the presence of *N*-methylimidazole (NMI), as evidenced by the synthesis of compound **48a** in Scheme 23.

In the first approach, the protected isopro-NR^+^OTf^−^ **29** was reacted with a 6-fold excess of phosphorus acid chloride in the presence of 8-fold excess of NMI. The purification by preparative TLC afforded the phosphorylated product **49** in 94% yield. The removal of the acetonide in 90% aqueous TFA was conducted at room temperature for 1 hour, and was followed by HPLC purification to yield compound **48a** in 15% yield. In the second approach, the mesitylene acetal protected NR^+^ was reacted with a 6-fold excess of *tert*-butylmagnesium bromide. The resulting magnesium alkoxide was treated with a 3-fold excess of phosphorus acid chloride. The crude mixture was purified by flash silica gel chromatography and further deprotected using HCl diluted in THF/MeOH mixture. Unfortunately, HPLC purifications of the final product **48a** (X = Cl) resulted in the isolation of this compound with only 1.5% yield recovery over two steps.

**Figure 11:**
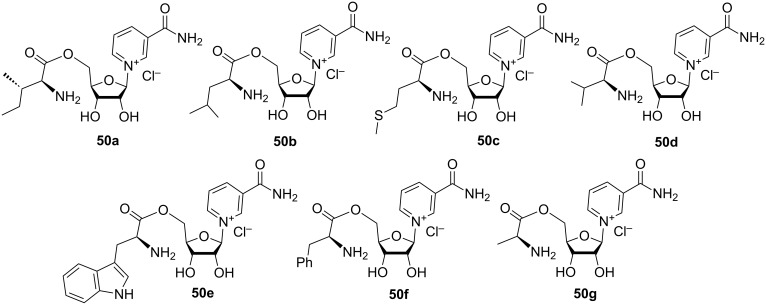
Structural formulas of amino acid NR^+^ conjugates.

**Scheme 24:**
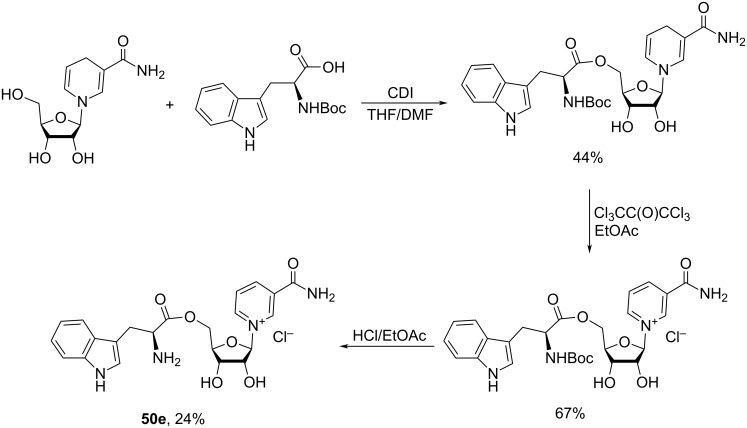
Synthesis of amino acid NR^+^ conjugates using NRH and protected amino acid under CDI-coupling conditions followed by re-oxidation with hexachloroacetone.

The patent publication of Migaud et al. [28] describes the preparation of conjugates **50a**–**g** of NR with amino acids such as isoleucine, leucine, methionine, valine, tryptophan, phenylalanine, and alanine (Figure 11). The synthesis of these conjugates is based on the carbonyl-diimidazole (CDI)-catalyzed coupling of amino acids with unprotected NRH, followed by oxidation of corresponding NRH–amino acid conjugates to the NR-conjugate derivatives, as illustrated by tryptophan conjugate **50e** in Scheme 24. According to the proposed methodology, Boc-protected amino acids were first treated with carbonyldiimidazole (CDI) in THF for several hours with the subsequent addition of a solution of NRH in DMF. The coupling reaction product was purified by silica gel column chromatography (6% MeOH in EtOAc as an eluent) and characterized by ^1^H NMR spectroscopy and high-resolution mass spectrometry data. If instead of CDI, the coupling between NRH and Boc-protected tryptophan was performed using HATU as coupling reagent in DIEA, the conjugate was obtained in only 5% yield. Oxidation of the 1,4-dihydropyridine core was performed using a ca. 9-fold excess of hexachloroacetone and required purification of the crude by silica gel column chromatography (30% MeOH in EtOAc as an eluent). The final step of the synthetic route consisted in the removal of the Boc protection by using a HCl/EtOAc mixture to yield the desired NR^+^ conjugate in 24% yield.

Overall, the data presented show that NR^+^ may be modified at the 5′-position using either direct reaction of NR^+^ with reactive electrophiles, such as (substituted) phosphorus acid chlorides, or via the intermediate reduction of NR^+^ to NRH. While successful, these reaction sequences remain low yielding and require cumbersome purification sequences, which reduce yields even further.

### 4. Synthesis of isotope-labelled analogues and derivatives of NR^+^

**Figure 12:**
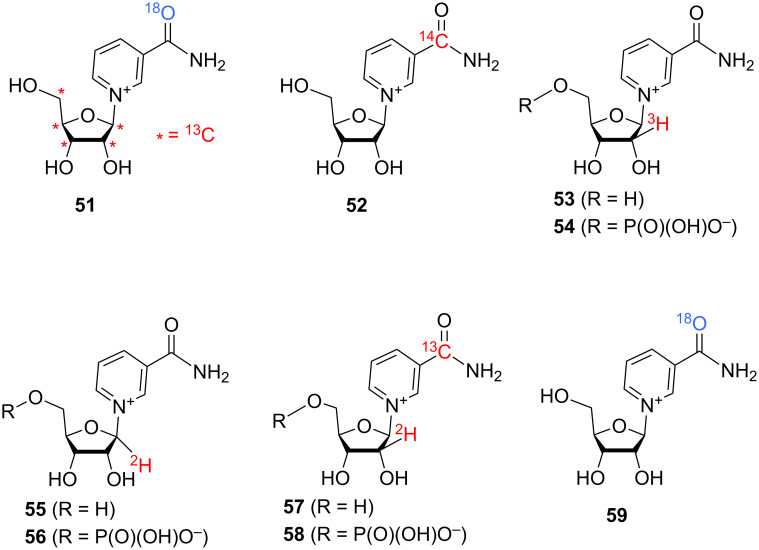
Chemical structures of known isotopically labelled NR^+^ analogues and derivatives.

Isotope-labelled analogues and derivatives of NR^+^ are very useful tools in studying various metabolic pathways which include complex and mutually dependent biochemical transformations of many nicotinoyl derivatives and related compounds in living cells. In the case of NR^+^ itself, isotope labels may be incorporated into the sugar residue (^2^H, ^3^H, ^18^O, ^13^C, and ^14^C isotopes) and the nicotinamide core (^18^O, ^13^C, ^14^C, and ^15^N isotopes). Figure 12 illustrates some currently known isotopomers of NR^+^ and NMN.

Generally speaking, there are two approaches available for the synthesis of isotopically labelled NR^+^ derivatives – a chemoenzymatic synthetic approach or a total synthesis approach.

The most common method for the enzymatic preparation of labelled NR^+^ consists in the enzyme-catalyzed degradation of the correspondingly labelled NAD^+^ or NMN derivative with the subsequent purification of the reaction products by chromatography on an ion exchange resin. A typical example of this methodology may be found in work of Kasasarov and Moat [44] describing preparation of [carbonyl-^14^C]NR^+^ **52** from the corresponding ^14^C-labelled NAD^+^ catalyzed by enzymes from *Proteus vulgaris* OX-19. Enzymatic approaches to isotope (^14^C, ^13^C, ^3^H, ^2^H, ^32^P, ^15^N (pyridinium nitrogen of NAD^+^)) labelled NAD^+^ or NMN are numerous and have been described in the related literature [65,87–93]. Those enzymatic methods make it possible to prepare not only NR^+^ derivatives labelled in the nicotinamide core but also in the ribosyl moiety if labelled phosphoribosyl pyrophosphate (PRPP) is used in the preparation of the dinucleotide.

The article of Saunders et al. [45] describes the preparation of [carbonyl-^14^C]NR^+^ **52** and of [4-^3^H]NR^+^ by treatment of the corresponding radio-labelled NMN with 5′-nucleotidase. The product was purified by ion exchange chromatography.

Recent advances in the chemo-enzymatic approach are illustrated in the work of Tran et al. [94], which describes the synthesis of [^13^C_5_,^18^O]-NR^+^ **51** (Figure 12). According to this approach, [U-^13^C]-glucose and nicotinic acid (NA) were enzymatically converted to ^13^C-NAAD containing a fully ^13^C-labelled ribosyl residue in the nicotinic acid riboside part of the NAAD molecule. This synthesis requires the usage of 10 enzymes (hexokinase, pyruvate kinase, glucose 6-phosphate dehydrogenase, glutamate dehydrogenase, 6-phosphogluconate dehydrogenase, phosphoriboisomerase, myokinase, nicotinate phosphoribosyltransferase, phosphoribosylpyrophosphate synthetase, and nicotinamide mononucleotide adenylyltransferase) along with ATP, phospho(enol)pyruvate (PEP), NADP^+^, α-ketoglutarate required for the enzymatic reactions. In the second – again enzymatic – step, the purified ^13^C-labelled NAAD was transformed to the corresponding ^13^C-NAD^+^ by NAD^+^ synthetase. Then, the purified ^13^C-NAD^+^ was incubated with ^18^O-Nam (prepared by chemical reaction of 3-cyanopyridine with ^18^O-water) in the presence of ADP-ribosylcyclase to yield ^13^C,^18^O-NAD^+^. The latter was subsequently enzymatically degraded using phosphodiesterase I and alkaline phosphatase to quantitatively afford ^13^C,^18^O-NR^+^. This publication also describes the enzymatic synthesis of ^14^C-NR^+^ **52** from unlabelled NAD^+^ and [carbonyl-^14^C]-Nam in the presence of ADP-ribosylcyclase to give ^14^C-NAD^+^, and subsequent treatment with phosphodiesterase I and alkaline phosphatase.

The above mentioned enzymatic synthesis relies mainly on works of Schramm et al. [95–98] which describe an enzymatic strategy for the synthesis of tritium and ^14^C-labelled NAD^+^, with the isotope labels being located in different positions of the ribosyl residue of the nicotinamide riboside moiety. The authors used [2-^3^H]-, [5-^3^H]-, [6-^3^H]-, [2-^14^C]-, and [6-^14^C]-glucose and nicotinic acid in enzymatic reactions to generate corresponding [1′-^3^H]-, [2′-^3^H]-, [4′-^3^H]-, [5′-^3^H]-, [1′-^14^C]-, [5′-^14^C]-NAD^+^. Using ^15^N-labelled nicotinic acid as a source of the label, the authors also prepared ^15^N-labelled NAD^+^ isotopomers, such as [1′-^14^C,1-^15^N]-NAD^+^ and [5′-^14^C,1-^15^N]-NAD^+^ (atomic locations in the ribosyl residue of NR^+^ part of NAD are designated by primed numbers).

The enzymatic synthesis of ^18^O-labelled NAD^+^ with the isotopic label incorporated as the amide oxygen of the nicotinamide moiety is described in the work of Yang et al. [99]. The authors converted enzymatically unlabelled NAD^+^ to labelled NAD^+^ using ^18^O-nicotinamide (20-fold excess) and glycohydrolase/cyclase CD38.

**Scheme 25:**
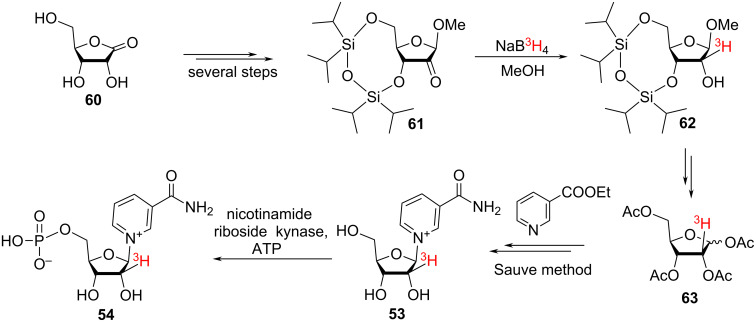
Synthesis of [2′-^3^H]-NR^+^ and [2′-^3^H]-NMN.

The chemical synthesis of tritium-labelled NR^+^ **53** and the subsequent enzymatic synthesis of the tritiated NMN **54** as well as the corresponding [2′-^3^H]-NAD^+^ is described in the work of Cen et al. [100] (Scheme 25).

The authors used ribonolactone **60** as a starting material and after multiple synthetic steps, they prepared the intermediate ketone **61**. The sequence included the protection of the 3,5-hydroxy moieties in **60** with an 1,1,3,3-tetraisopropyldisiloxanylidene group, the conversion of the ketone function into a MeO-group by reaction with DIBAL-H followed by quenching with methanol. This was followed by the oxidation of the hydroxy group at the 2-position of the ribosyl moiety by pyridinium chlorochromate to yield the ketone **61**. This key synthetic intermediate ketone was reduced using sodium borotritide to incorporate the tritium label on the ribose unit. Removal of the siloxanyl protection from **62** and conversion of all the hydroxy functions and the methoxy group into acetates resulted in 2-^3^H-labelled ribose tetraacetate **63** that was glycosylated with ethyl nicotinate following the Sauve procedure (see Scheme 10 above) to yield the 2′-^3^H-labelled NR^+^ **53**.

The above work also describes synthesis of NAD^+^ containing ^18^O-label in the NR^+^ portion of the molecule and originating from [5-^18^O]-glucose [100].

**Scheme 26:**
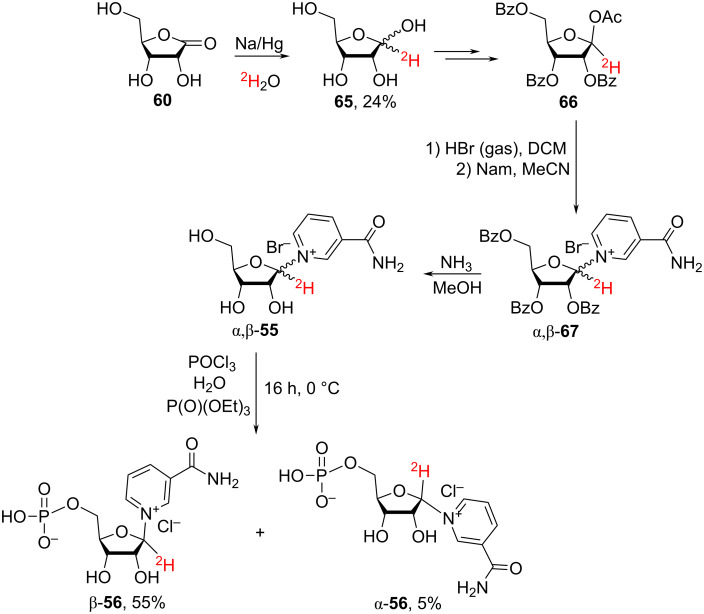
Synthesis of α- and β-anomers of [1′-^2^H]-NMN.

The work by Bull et al. [101] describes the chemical synthesis of the deuterium-labelled [1′-^2^H]-NR^+^Br^−^ **55** and [1′-^2^H]-NMN **56** (see Figure 12 and Scheme 26).

In the first step of the synthesis, the ribonolactone **60** was reduced to [1-^2^H]-ribose **65** using sodium amalgam and D_2_O. Subsequently, ^2^H-labelled ribose **65** was converted to 1-*O*-acetyl-2,3,5-tri-*O*-benzoyl-β-D-[1-^2^H]-ribofuranose that was then converted to 2,3,5-tri-*O*-benzoyl-D-[1-^2^H]-ribofuranosyl bromide and used for the glycosylation of Nam according to the procedure described by Jarman et al. [22] to furnish – after removal of benzoyl groups – the 3-carbamoyl-1-(D-[1′-^2^H]-ribofuranosyl]pyridinium bromide **55**. The bromide salt was then used, without additional purification, for the synthesis of [1′-^2^H]-NMN **56**. The pure β-anomer of **56** was obtained after column chromatography on AG-50W-X8 cation-exchange resin and subsequently used to enzymatically prepare the corresponding [1′-^2^H]-NAD^+^ that was converted to [carbonyl-^14^C,1′-^2^H]-NAD^+^ by means of an enzyme-catalyzed exchange with [carbonyl-^14^C]-Nam.

Mills et al. [102] mention the use of the double-labelled NMN **58** in mass spectrometry studies. This doubly labelled NMN was obtained according to the procedure described in work of Lee et al. [27] starting from corresponding doubly labelled [2′-^2^H,carbonyl-^13^C]-NR^+^ **57**. Compound **57** was prepared from the corresponding 1,2,3,5-tetra-*O*-acetyl-β-D-[2-^2^H]-ribofuranose and [carbonyl-^13^C]-Nam according to the Vorbrüggen methodology described by Ratajczak et al. [103] and Trammell et al. [104]. These two works also mention the synthesis of ^18^O-labelled NR^+^ **59** from ^18^O-Nam; subsequent synthesis of ^18^O-labelled NMN by phosphorylation of compound **59** with NRK1 is described in the work of Ratajczak et al. [103].

## Conclusion

On the one hand, this review demonstrates the recent achievements in the efficient synthesis of the anomerically pure β-form of nicotinamide riboside salts (NR^+^X^−^) and of the analogues, derivatives and conjugates of NR^+^, including those containing isotopic labels. On the other hand, this review also highlights the fact that there are still many synthetic challenges to be addressed. As such, a better understanding of the chemical versatility of the ribosylated forms of the nicotinoyl moiety will offer more reliable, more scalable and more reproducible preparations of NR^+^X^−^ salts. Furthermore, a better understanding of the reactivity of the ribosylated scaffold will enable more effective modifications of the riboside residue of NR^+^ at the 5′-hydroxy position, so to ensure higher yields, better recovery and improved purification strategies. This will enable the improved preparation of new forms of pharmaceutically acceptable and potentially useful forms of NR^+^ in addition to the atom-efficient syntheses of isotopically labelled NR^+^ analogues and derivatives.
